# Nociceptin/Orphanin FQ Peptide (NOP) Receptor Agonists as Novel Analgesics in Pain Medicine

**DOI:** 10.3390/jcm15207942

**Published:** 2026-10-14

**Authors:** Islam Mohammad Shehata, Manar Ahmed Kamal, Taha Muhammad Makki, Basant Mohamed Salah Eldin, Sandra Kareem Basta, Cathrin Amgad Abdelmalak, Zeyad Rashed, Abdul-rahman A. Hegazi, Ahmed I. Anwar, Danielle D. Ward, Alan D. Kaye

**Affiliations:** 1 Department of Anesthesia, Faculty of Medicine, Ain Shams University, Cairo 11566, Egypt; 2 Mit Ghamr Nephrology and Urology Hospital (MNUH), Mansoura 35612, Egypt; 3 Faculty of Medicine, Benha University, Benha 13511, Egypt; 4 Faculty of Medicine, Modern University for Technology and Information, Cairo 11585, Egypt; 5 Department of Anesthesiology, LSU Health Shreveport, Shreveport, LA 71103, USA; 6 Adneuris Therapeutics, Monmouth Junction, NJ 08852, USA

**Keywords:** nociceptin, orphanin, emerging analgesics, dual NOP/MOP agonists, cebranopadol, pain, spinal cord, opioid receptor, β-arrestin

## Abstract

Chronic pain is a major cause of disability worldwide, impacting around 20% of adults globally. Conventional mu-opioid peptide (MOP) receptor agonists provide effective analgesia; however, their prolonged use is restricted by severe dose-limiting toxicities such as opioid-induced respiratory depression, analgesic tolerance, hyperalgesia, physical dependence, and addiction. The goal of this narrative literature review was to compile the available data on NOP receptor-targeted analgesics, with a focus on dual NOP/MOP agonists and cebranopadol. The pharmacological mechanisms, preclinical and clinical evidence, safety profile, and potential therapeutic role in pain management were all highlighted. The nociceptin/orphanin FQ (N/OFQ) peptide (NOP) receptor system has been identified as a new pathway for managing pain through mechanisms that differ from those of classical opioid signaling. The NOP receptor is a Class A G-protein-coupled receptor (GPCR) that preferentially couples to inhibitory G(i/o) proteins. Upon activation, it suppresses adenylyl cyclase activity, lowers intracellular cyclic adenosine monophosphate (cAMP) levels, blocks voltage-gated calcium channels, and activates G-protein-gated inwardly rectifying potassium channels, thereby hyperpolarizing neurons and decreasing excitatory neurotransmitter release. NOP receptors are anatomically distributed throughout the peripheral and central nervous systems, including primary afferent sensory neurons, dorsal root ganglia, spinal dorsal horn, periaqueductal gray, rostral ventromedial medulla, central amygdala, ventral tegmental area, and nucleus accumbens. NOP activation via these pathways offers significant pain relief in acute nociception, as well as in chronic inflammatory and neuropathic pain conditions. Supraspinal activation of NOP within the mesocorticolimbic pathway influences the emotional aspect of pain by reducing corticotropin-releasing factor signaling and decreasing mesolimbic dopamine release. NOP agonism effectively provides analgesia while also attenuating drug reward and reinforcement. The development of pharmaceuticals utilizing NOP agonism has progressed from endogenous peptide analogues to selective non-peptide agonists, and more recently to bifunctional NOP/MOP (dual NMR) agonists, such as the dual NOP/MOP agonist cebranopadol, which has passed Phase I–III clinical testing. There are still significant questions about long-term safety, comparative efficacy, and the interpretation of unpublished or preliminary Phase III results, despite the fact that available clinical trials indicate analgesic efficacy with perhaps decreased respiratory and abuse-related effects. This drug achieves equianalgesic efficacy through potent dual NOP/MOP activation while significantly reducing the risk of respiratory depression and drug-liking. However, clinical translation of NOP receptor agonism is hindered by several challenges, including supraspinal pronociceptive paradoxes, species-specific pharmacology, dose-dependent sedation, and regulatory complexities associated with mixed-mechanism ligands. NOP-targeted therapies represent a promising approach for the development of next-generation, opioid-sparing analgesics in pain medicine.

## 1. Introduction

Pain is one of the leading causes of disability worldwide and affects hundreds of millions of individuals, imposing substantial medical, social, and economic burdens. Chronic pain alone affects approximately 20% of adults globally and remains inadequately controlled in many patients despite advances in multimodal analgesia [1,2].

Despite proven efficacy in the management of moderate-to-severe pain, conventional μ-opioid peptide (MOP) receptor agonists are associated with several important clinical limitations, including respiratory depression, tolerance, dependence, opioid-induced hyperalgesia, and misuse, which collectively restrict their long-term therapeutic utility [3]. Opioid-induced respiratory depression (OIRD) occurs in approximately 0.5–2% of postoperative patients receiving opioid analgesics [4]. Long-term opioid therapy is associated with opioid misuse in 21–29% of patients and opioid addiction (opioid use disorder) in 8–12% of patients [5]. These limitations have prompted the investigation of alternative analgesic targets capable of maintaining effective pain relief while minimizing opioid-related toxicities [5,6].

Among the new non-classical opioid targets, the nociceptin/orphanin FQ (N/OFQ) receptor, also known as the nociceptin opioid peptide (NOP) receptor, system has attracted considerable attention because it modulates nociceptive processing through mechanisms that differ from those of classical opioid receptors [6,7]. Preclinical and early clinical studies suggest that NOP receptor activation provides effective analgesia, particularly in inflammatory and neuropathic pain states, while producing less respiratory depression, lower abuse liability, and reduced development of tolerance compared with selective MOP agonists [6,7]. Furthermore, new evidence suggests that NOP receptor activation can modulate MOP-mediated adverse effects, making selective NOP agonists and bifunctional NOP/MOP receptor (dual NMR) agonist ligands attractive candidates for the development of safer next-generation analgesics [7,8].

To better understand the therapeutic potential of this system, it is essential to consider the biological characteristics of its endogenous ligand and receptor. Nociceptin/orphanin FQ (N/OFQ) is a 17-amino acid endogenous neuropeptide derived from the precursor protein prepronociceptin (PNOC). It was independently identified in 1995 as the endogenous ligand for the nociceptin opioid peptide (NOP) receptor, previously known as opioid receptor-like 1 (ORL1) [9,10]. Although N/OFQ shares considerable structural homology with endogenous opioid peptides, particularly dynorphin A, it exhibits distinct pharmacological characteristics because it selectively binds to the NOP receptor rather than the classical opioid receptors, namely the μ-opioid peptide (MOP) receptor, δ-opioid peptide (DOP) receptor and κ-opioid peptide (KOP) receptor [6]. This unique receptor selectivity distinguishes the N/OFQ–NOP receptor system from classical opioid signaling and provides the biological basis for its expanding therapeutic potential in pain modulation, anesthesia, and other neurological disorders [6,7].

Given the growing interest in NOP-targeted analgesics, this review aims to provide a clear and critical overview of the N/OFQ–NOP receptor system and its potential role in pain management. It discusses the molecular biology and pharmacological features of the NOP receptor, its involvement in pain processing, and the development of selective NOP agonists and bifunctional NOP/MOP receptor ligands. The review also examines the available preclinical and clinical evidence on their analgesic effects and safety, with particular emphasis on cebranopadol, before discussing the functional implication, therapeutic application, current limitation, translational challenges and future directions of NOP-targeted analgesic development.

## 2. NOP Receptor Biology and Pharmacology

### 2.1. Receptor Identity and Molecular Features

The nociceptin opioid peptide (NOP) receptor, previously known as opioid receptor-like 1 (ORL1), is the fourth member of the opioid receptor family and is classified within the class A (rhodopsin-like) G-protein-coupled receptor (GPCR) superfamily. Although NOP shares approximately 60% amino acid sequence homology with the classical μ-(MOP), δ-(DOP), and κ-opioid (KOP) receptors, it possesses distinct pharmacological properties that justify its classification as an independent opioid receptor subtype rather than a classical opioid receptor [6,9,10,11].

The NOP receptor was identified in 1994 prior to its endogenous ligand being known, which led to its initial designation as an orphan receptor. Following this, two independent groups discovered nociceptin/orphanin FQ (N/OFQ), a 17-amino acid peptide originating from prepronociceptin (PNOC), to be the endogenous ligand for NOP [9,10]. Unlike endogenous opioid peptides such as endorphins, enkephalins, and dynorphins, N/OFQ exhibits negligible affinity for MOP, DOP, and KOP, while classical opioid ligands—including morphine, fentanyl, naloxone, and naltrexone—display minimal affinity for the NOP receptor [7,10]. The unique ligand selectivity is the basis for the distinct physiological functions of the N/OFQ–NOP receptor signaling system.

**Figure 1:**
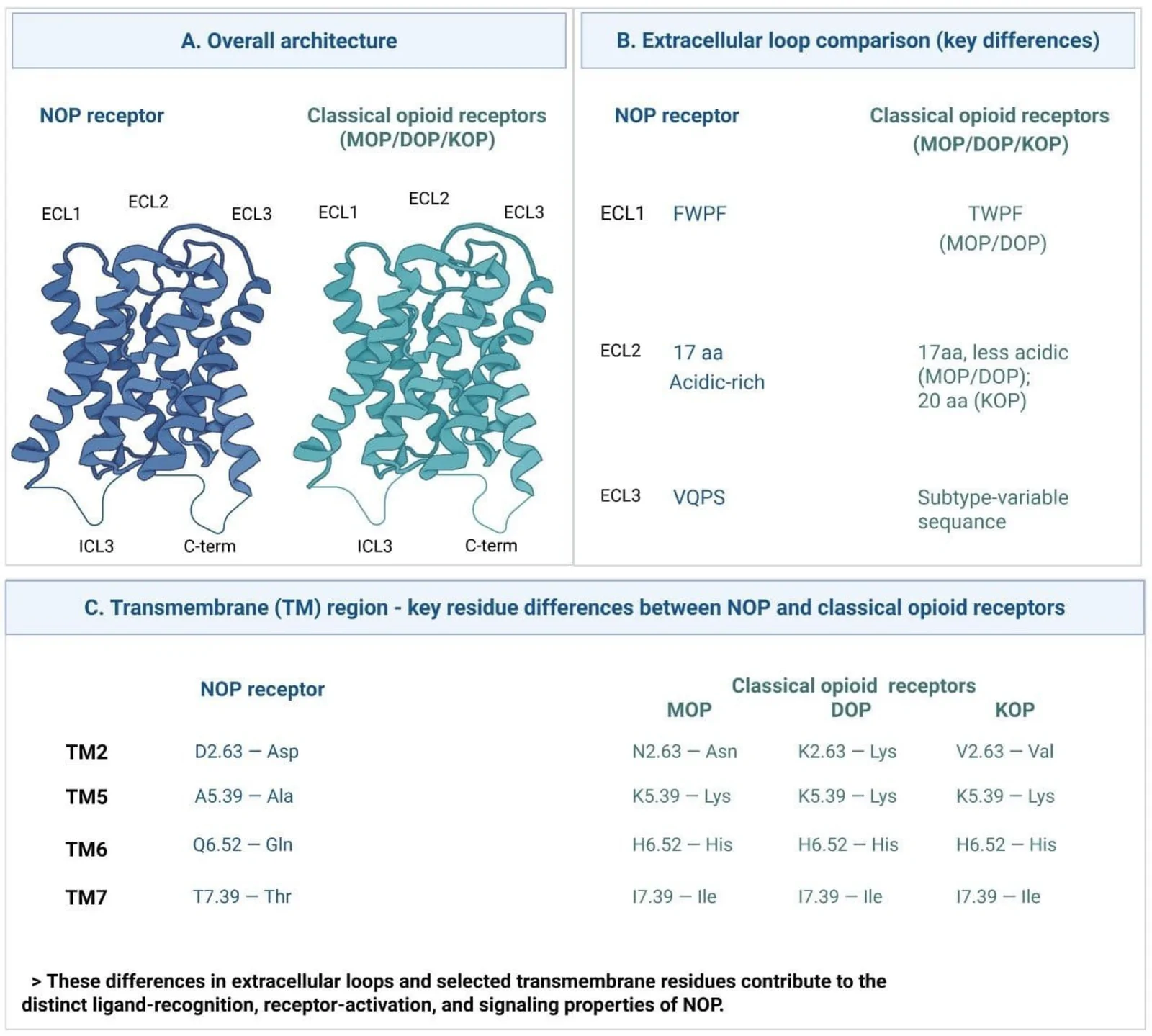
Structural determinants and molecular architecture distinguishing the NOP receptor from classical opioid receptors. Comparative structural analysis illustrating the key molecular differences between the NOP receptor and classical opioid receptors. Panel (**A**) highlights the overall seven-transmembrane architecture and extracellular/intracellular loop arrangements. Panel (**B**) compares extracellular loop (ECL) sequences, noting the shorter ECL2 and conserved Cys-Phe-Cys/W-G-P motifs in the NOP receptor. Panel (**C**) outlines critical transmembrane amino acid residue variations across TM1–TM7 [13,14]. Created in BioRender. ElSayed, M. (2026) https://BioRender.com/q184lwk (accessed on 30 August 2026).

The NOP receptor has a characteristic seven-transmembrane helical structure typical of GPCRs, with its extracellular domains involved in ligand recognition and its intracellular domains responsible for G-protein coupling (Figure 1). Subtle differences within the extracellular binding pocket of the receptor account for its selective recognition of N/OFQ and synthetic NOP agonists, despite its structural similarity to classical opioid receptors. Advances in molecular pharmacology and computational modelling have further identified key amino acid residues that govern ligand binding, receptor activation, and functional selectivity, facilitating the rational design of novel NOP-targeted therapeutics [6,12].

The pharmacological profile of the NOP receptor is significantly different from that of conventional opioid receptors (Table 1). The activation of the mu opioid receptor primarily mediates supraspinal analgesia but is also responsible for respiratory depression, euphoria, constipation, and physical dependence. Activation of NOP receptors typically results in analgesia with a lower risk of respiratory depression and abuse potential, especially in cases of chronic inflammatory and neuropathic pain. Significant interest has been sparked in selective NOP agonists and bifunctional NOP/MOP ligands as safer alternatives to traditional opioid analgesics [6,15].

### 2.2. Neuroanatomical Distribution

**Figure 2:**
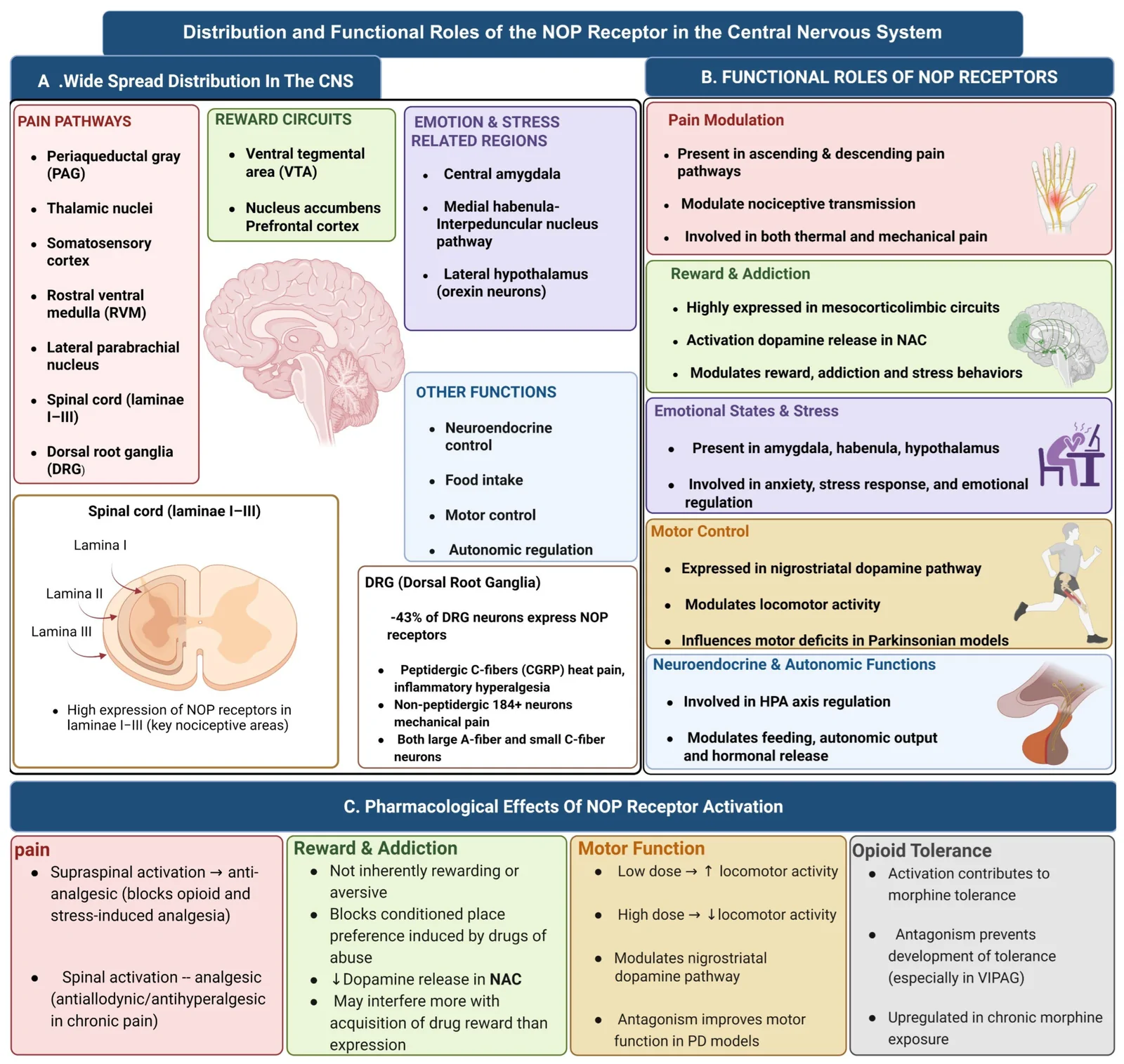
Neuroanatomical distribution and functional roles of the NOP receptor across central pain and reward pathways. Schematic representation detailing the anatomical localization and biological functions of NOP receptors throughout the central and peripheral nervous systems. The diagram summarizes NOP-mediated modulation across nociceptive processing, mesolimbic reward circuits, emotional/stress states, motor control, and neuroendocrine regulation. Colors are used for visual organization to help readers distinguish and relate the different functional categories; they do not represent specific biological properties or relationships. Upward arrows (↑) indicate an increase, whereas downward arrows (↓) indicate a decrease in the corresponding measure or activity. Created in BioRender. ElSayed, M. (2026) https://BioRender.com/vwk6xsc (accessed on 30 August 2026). Schematic representation of NOP receptor activation by nociceptin/orphanin FQ (N/OFQ) leading to G-protein dissociation and engagement of downstream intracellular signaling pathways that contribute to antinociceptive effects. Created in BioRender. ElSayed, M. (2026) https://BioRender.com/b17mul2 (accessed on 30 August 2026).

The widespread distribution of the NOP receptor throughout the central and peripheral nervous systems explains its diverse physiological roles (Figure 2).

The superficial laminae of the spinal dorsal horn, dorsal root ganglia (DRG), periaqueductal gray (PAG), rostral ventromedial medulla (RVM), thalamus, amygdala, hippocampus, hypothalamus, and cerebral cortex have been shown to highly express NOP receptors, as have peripheral sensory neurons and immune cells [23,24,25]. In the spinal cord, NOP receptors are highly expressed in nociceptive pathways where they regulate primary afferent neurotransmission. Activation of spinal NOP receptors inhibits the release of excitatory neurotransmitters such as glutamate and substance P, thereby reducing synaptic transmission from primary afferent neurons to second-order dorsal horn neurons. These mechanisms contribute to the pronounced antinociceptive effects seen in models of inflammatory and neuropathic pain [15,23].

The NOP receptor is widely distributed in supraspinal regions involved in descending pain modulation. Receptor activation in the PAG and RVM alters descending inhibitory and facilitatory pathways in a state-dependent manner, yielding either pronociceptive or antinociceptive responses that depend on the site of administration, pain model, and species studied. The context-dependent regulation that characterizes the NOP receptor differentiates it from classical opioid receptors and underscores the complexity of pain modulation mediated by NOP [6,15].

Experimental studies demonstrate that activation of NOP receptors suppresses mesolimbic dopamine release and attenuates rewarding effects of opioids, alcohol, and other drugs of abuse, suggesting an important role in reducing abuse liability while preserving analgesic efficacy [25,26].

### 2.3. Intracellular Signaling and Functional Output

**Figure 3:**
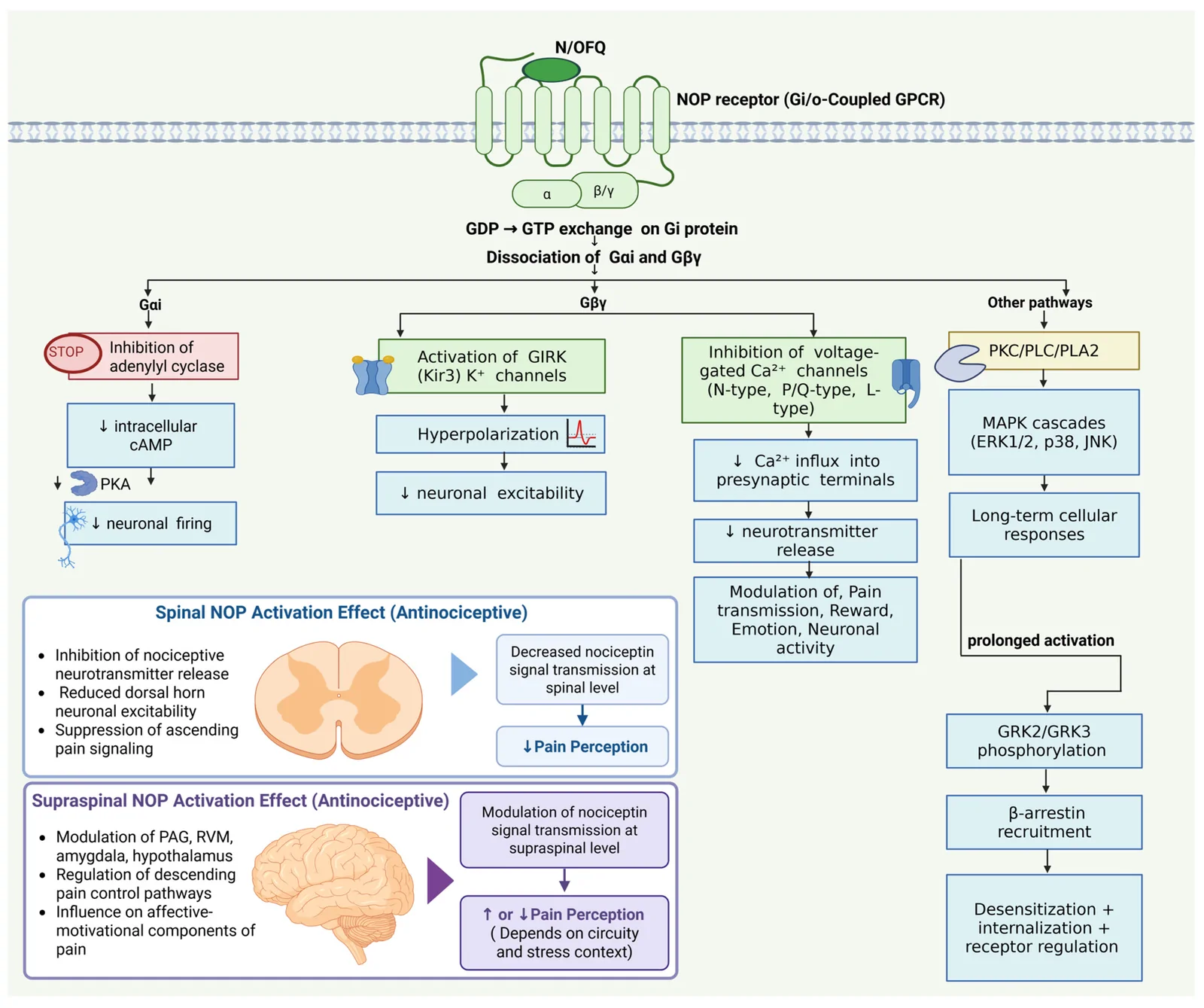
Canonical intracellular signaling pathways activated by the nociceptin/orphanin FQ (NOP) receptor. The colors distinguish the different signaling pathways: Gβγ is shown in green, Gαi in red, and other pathways in yellow, while their downstream effects are shown in blue. Arrows indicate the direction and sequence of signaling events, and downward-pointing arrows that do not point to a box indicate a decrease in the corresponding component or effect.

Following ligand binding, the NOP receptor primarily couples to inhibitory Gi/o proteins, leading to downstream intracellular signaling that modulates neuronal activity (Figure 3).

Activation of the NOP receptor also regulates multiple ion channels that govern synaptic transmission. This includes inhibition of voltage-gated calcium channels, reducing calcium influx and decreasing the release of excitatory neurotransmitters such as glutamate, substance P, and calcitonin gene-related peptide (CGRP). At the same time, activation of G-protein-gated inwardly rectifying potassium (GIRK) channels increases potassium efflux, causing neuronal hyperpolarization and a decrease in action potential transmission. These mechanisms combined reduce pain signaling at both spinal and supraspinal levels [6,15,27] (Figure 3).

**Figure 4:**
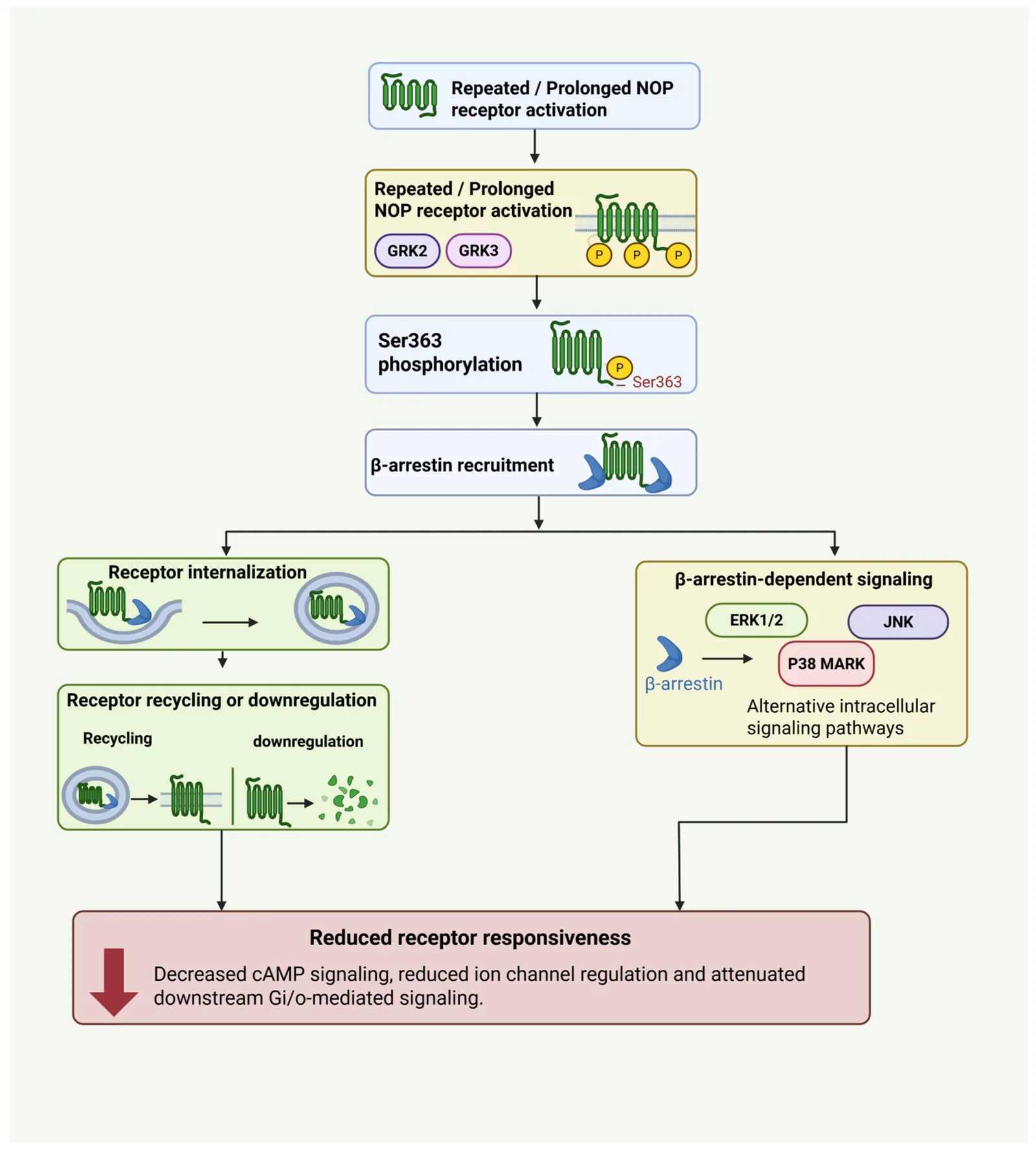
Mechanisms of NOP receptor regulation, desensitization, and trafficking following prolonged agonist exposure. Prolonged NOP receptor activation triggers regulatory mechanisms that govern receptor desensitization and signaling responsiveness. Arrows indicate the direction and sequence of the molecular interactions and cellular processes depicted. The right-pointing arrow in the receptor internalization panel indicates the progression of the receptor internalization process. The right-pointing arrow in the β-arrestin-dependent signaling panel indicates the direction of signaling toward the downstream MAPK pathways (ERK1/2, p38 MAPK, and JNK); however, the involvement of β-arrestin in regulating individual MAPK pathways may vary. The phosphorylation symbol (P) indicates receptor phosphorylation. Different colors are used solely for visual distinction and readability and do not represent specific molecular properties or functional differences.Created in BioRender. ElSayed, M. (2026) https://BioRender.com/jv1kvaj (accessed on 30 August 2026).

Prolonged activation of the NOP receptor can recruit β-arrestins and stimulate several downstream kinase pathways (Figure 4), including ERK, JNK, and p38 MAPK, beyond the conventional Gi/o-mediated signaling pathway. Signaling cascades contribute to receptor desensitization, internalization, and longer-term modulation of neuronal plasticity. Growing evidence suggests that biased NOP agonists can selectively activate G-protein-dependent pathways while limiting β-arrestin recruitment, which may help maintain analgesic efficacy and decrease adverse effects associated with prolonged receptor activation [12,27].

The physiological effects of NOP receptor activation are heavily influenced by both the specific anatomical site and the underlying pathological condition (Table 2). Activation of the NOP receptor consistently yields robust antinociceptive effects under conditions of chronic inflammation and neuropathic pain, whereas its actions in acute pain models are more variable and may depend on the site of receptor activation. The regulation of reward processing by the NOP receptor is believed to contribute to reduced abuse liability with NOP-targeted analgesics compared to conventional μ-opioid receptor agonists. These properties support the development of selective NOP agonists and bifunctional NOP/MOP ligands as promising next-generation analgesics [15,24,25].

## 3. NOP-Targeting Ligands and Drug Development

### 3.1. Classes of NOP-Active Compounds

Since the discovery of nociceptin/orphanin FQ (N/OFQ) and its receptor in the mid-1990s, considerable efforts have focused on developing ligands capable of leveraging the therapeutic potential of the nociceptin opioid peptide (NOP) receptor for pain management [10]. Research into drug development has advanced from endogenous peptide analogues to highly selective non-peptide agonists, and it has also recently focused on bifunctional ligands that target NOP/μ-opioid peptide (MOP) receptors known as dual NMR agonists. This new focus on dual NMR agonism resulted in the development of cebranopadol, which is currently the most advanced NOP-targeted analgesic being investigated clinically [36]. NOP-targeting ligands can be classified based on their chemical structure (peptide or non-peptide), route of administration, or pharmacological properties. They are categorized in this review according to their pharmacological profile as selective NOP agonists, mixed NOP/MOP agonists, and NOP antagonists (Table 3), as this classification best reflects their therapeutic application in pain management [37].

#### 3.1.1. Selective NOP Agonists

Selective NOP receptor agonists were developed to take advantage of the analgesic effects of NOP receptor activation while avoiding the adverse effects linked to classical MOP agonists. Several peptide-based agonists, including UFP-112 and PWT2-N/OFQ, were developed as prototypes based on the endogenous peptide nociceptin/orphanin FQ (N/OFQ), which exhibited potent antinociceptive activity in preclinical models [42]. Although N/OFQ shares structural similarities with endogenous opioid peptides, it exhibits negligible affinity for the classical opioid receptors (MOP, DOP, and KOP), binding selectively to the NOP receptor instead [43]. Consequently, selective NOP agonists can offer analgesia with a decreased risk of respiratory depression, physical dependence, and abuse liability compared to traditional opioids [38].

Substantial efforts were directed towards the development of selective non-peptide NOP agonists to overcome the poor pharmacokinetic properties of peptide ligands. Ro 64-6198 was the initial highly selective compound, boasting more than a 100-fold preference for the NOP receptor compared with classical opioid receptors [44,45]. Studies on Ro 64-6198 showed that the pain-reducing effects were removed by selective NOP antagonist J-113397 but not by MOP antagonist naltrexone, suggesting that its analgesia was through the activation of NOP receptors only [46]. Nevertheless, despite favorable central nervous system penetration, the clinical development of Ro 64-6198 was limited by poor oral bioavailability and dose-dependent adverse effects, including sedation and hypothermia in pre-clinical studies [47,48].

Selective non-peptide NOP agonist SCH 221510 showed significant antinociceptive efficacy in non-human primate models, indicating the potential for translating selective NOP receptor activation clinically [49,50]. However, relatively low oral bioavailability and short elimination half-life substantially limited suitability for clinical development [51]. These findings collectively indicate that while selective NOP agonists offer valuable proof-of-concept for NOP-mediated analgesia, their pharmacokinetic limitations and incomplete efficacy have restricted their clinical translation.

#### 3.1.2. Mixed NOP/MOP Agonist

Although selective NOP agonists demonstrated encouraging analgesic activity in experimental studies, their clinical utility has been limited by modest efficacy in certain pain models and adverse effects such as sedation and motor impairment [52,53]. These limitations prompted the development of bifunctional ligands that can simultaneously activate both NOP and MOP receptors.

The rationale for dual receptor activation is based on harnessing the complementary pharmacological properties of both receptors. Activation of MOP remains one of the most effective mechanisms for producing potent analgesia, while concurrent activation of NOP receptors may attenuate several opioid-related adverse effects, including respiratory depression, reward, tolerance, and physical dependence. Activating both receptor systems simultaneously targets multiple neurobiological pathways involved in acute and chronic pain, potentially offering broader and more sustained analgesic efficacy than single-receptor activation [54,55].

Bifunctional ligands such as [Dmt^1^]N/OFQ(1–13)-NH_2_, PWT2-[Dmt^1^]N/OFQ(1–13)-NH_2_, BU08028, BU10038, AT-121, SR14150, SR16435, and Cebranopadol have shown promising preclinical efficacy [39]. Cebranopadol has progressed the furthest in clinical development among these compounds and currently serves as the leading candidate for translating dual NMR agonist pharmacology into clinical pain management.

#### 3.1.3. NOP Antagonists

In contrast to NOP agonists and mixed NOP/MOP agonists, NOP antagonists remain relatively underdeveloped for pain management. Use of NOP antagonists, such as J-113397, has been primarily limited to confirming agonist properties of NOP receptor ligands. However, NOP antagonists can induce anti-nociception at specific doses in rodent models, as demonstrated by supraspinal administration of 10 nmol UFP-101 in the mouse tail withdrawal assay [56]. In addition, they could potentially be used to treat side effects of NOP agonism such as sedation, similar to how naloxone is used in opioid-induced respiratory depression, although this therapeutic applicability is not fully established [57].

Beyond pain management, preclinical studies suggest that NOP antagonists have potential for other indications due to antidepressant and anxiolytic effects [58]. BTRX-246040 (LY 2940094), a selective NOP receptor antagonist, has demonstrated favorable pharmacokinetics (oral bioavailability exceeding 60%, a half-life of 6–12 h, along with CNS penetration) in Phase II trials for major depressive disorder and alcohol use disorder [59]. These findings demonstrate that, although NOP antagonists have not progressed like other classes for analgesic purposes, they remain relevant as research tools and as potential therapies for other indications.

#### 3.1.4. Functional Selectivity

Biased agonism, also known as functional selectivity, has emerged as an important concept in G-protein-coupled receptor pharmacology. Instead of activating all intracellular signaling pathways to the same extent, biased ligands preferentially stimulate specific signaling cascades, thereby potentially enhancing therapeutic efficacy while minimizing adverse effects [37,60].

Traditionally, analgesic effects of opioid receptors have been attributed primarily to G-protein-mediated signaling, whereas β-arrestin recruitment has been implicated in receptor desensitization, tolerance, and several opioid-related adverse effects [61]. This concept has driven the development of ligands that preferentially activate G protein signaling while limiting β-arrestin recruitment.

Compounds such as oliceridine, PZM21, and SR-17018 were initially identified as G-protein-biased MOP agonists with enhanced safety profiles. Subsequent investigations indicated that their favorable pharmacological characteristics are largely a result of reduced intrinsic efficacy rather than true signaling bias [37]. The precise contribution of biased agonism to opioid safety remains an area of ongoing investigation, necessitating further research to establish whether signaling bias can reliably translate into clinically significant improvements in analgesic therapy [62].

### 3.2. Cebranopadol

Among currently available bifunctional ligands, Cebranopadol represents the most advanced NOP-targeted analgesic. This first-in-class analgesic exhibits high affinity for both NOP and MOP while also interacting with κ- and δ-opioid receptors to a much lesser extent, thereby producing potent and long-lasting analgesia across multiple preclinical pain models [63]. The most clinically advanced NOP/MOP-targeted analgesic cebranopadol has passed Phase I and Phase II clinical assessment. Phase III investigations in acute postoperative pain have since been carried out. However, the lack of complete peer-reviewed primary publications of the ALLEVIATE-1 and ALLEVIATE-2 trials continues to restrict the interpretation of its Phase III effectiveness. As a result, its clinical development should be seen as promising, but it still has to be confirmed by comprehensive, peer-reviewed safety and effectiveness data. Table 4 summarizes the chronological development of Cebranopadol from receptor characterization and preclinical evaluation through clinical trials, illustrating its progression from molecular discovery to advanced clinical investigation. Cebranopadol’s most common reported side effect is nausea [64], which was described as the predominant treatment-emergent adverse event in the cebranopadol Phase 3 trials ALLEVIATE-1 and ALLEVIATE-2.

As concluded from the table above regarding the clinical efficacy and safety profile of Cebranopadol, the mentioned early clinical trials indicate that the drug is safe and effective in the treatment of patients with various pain conditions such as chronic osteoarthritis, diabetic peripheral neuropathy, acute postoperative pain, low back pain, and cancer-related moderate-to-severe pain [65,66,67].

The Pooled Analysis of Clinical Opiate Withdrawal Scales (COWS) of the previously mentioned five phase 2 trials concluded that the abrupt discontinuation of cebranopadol in phase 2 trials resulted in limited physical dependence and minimal withdrawal effects. The incidence of withdrawal was lower than that of opioid comparators, and withdrawal symptoms exceeding mild severity showed no difference from those of placebo. With supratherapeutic doses, physical dependence remained low, supporting cebranopadol’s safety profile [68].

Regarding human abuse potential and respiratory safety, cebranopadol demonstrates lower abuse potential and reduced respiratory depression at therapeutic doses than conventional MOP-selective opioids, with the lower abuse potential being consistent with NOP receptor activation opposing the mesolimbic reward pathway described in Section 4 [69,70].

**Table 4:** Translational profile and clinical development pipeline of cebranopadol.

Developmental Phase	Title	Model/Population	Intervention	Key Findings	Comments
Discovery	Discovery of a Potent Analgesic NOP and Opioid Receptor Agonist: Cebranopadol [36]	-	-	Cebranopadol (GRT-6005) identified as potent dual NOP/MOP full agonist; adequate oral bioavailability and CNS penetration confirmed	Medicinal chemistry paper
	Cebranopadol: A Novel Potent Analgesic Nociceptin/Orphanin FQ Peptide and Opioid Receptor Agonist [8]	-	Human recombinant NOP, MOP, KOP, and DOP receptors stably expressed in CHO cell membranes	Full agonist: NOP Ki ≈ 0.9 nM, MOP Ki ≈ 0.7 nM, KOP Ki ≈ 2.6 nM, DOP Ki ≈ 18 nM; Emax comparable to endogenous reference ligands at all four receptors	Introduction of Cebranopadol to the research world
In vitro	Pharmacological characterization of Cebranopadol a novel analgesic acting as mixed nociceptin/orphanin FQ and opioid receptor agonist [71]	-	Cebranopadol evaluated using calcium mobilization and BRET assays to characterize receptor activation and its specificity in G-protein and β-arrestin coupling assays	NOP = MOP > KOP ≥ DOP in calcium mobilization; G-protein preferential coupling at NOP and MOP; markedly reduced β-arrestin 2 recruitment relative to G-protein coupling, most pronounced at NOP	Confirmed pharmacology of Linz 2014 and gave insight into biased agonism
	Cebranopadol: A Novel Potent Analgesic Nociceptin/Orphanin FQ Peptide and Opioid Receptor Agonist [8]	-	Human MOP, DOP, KOP, and NOP receptor binding assays were run in microtiter plates	Cebranopadol showed the most pronounced binding affinities at human NOP and MOP receptors with sub nanomolar inhibitory constants, ∼3- to 4-fold weaker binding affinity in a human KOP receptor binding assay	Established Cebranopadol as a potent dual NOP/MOP receptor agonist with a pharmacological profile suitable for analgesic development.
Rodent	Cebranopadol: A Novel Potent Analgesic Nociceptin/Orphanin FQ Peptide and Opioid Receptor Agonist [8]	Tail-Flick Model CFA-Induced Arthritis Inflammatory Pain Bone Cancer Pain Model	Cebranopadol across multiple rat pain models at 0.2–21 µg/kg i.v. and up to 30.4 µg/kg p.o.	Potent antinociception across all models; ED50 tail flick = 5.6 µg/kg i.v. (~200× more potent than morphine); effect attenuated by both naloxone (MOP antagonist) and J-113397 (NOP antagonist), confirming dual NOP + MOP contribution in vivo	-
		CCI rat model tolerance profile	Cebranopadol once daily i.p. at analgesic dose for up to 26 days vs. morphine once daily i.p.	Complete tolerance to cebranopadol by day 22–26 vs. day 11 for morphine, approximately 2× longer time to full tolerance development	-
		Whole-Body Plethysmography	Intravenous administration of vehicle or 4, 8, or 16 µg/kg cebranopadol	No significant respiratory parameter changes at any cebranopadol dose, including the highest tested (16 µg/kg i.v.); morphine produced dose-dependent, profound respiratory depression under the same conditions	-
		-	-	half-life of 4.5 h oral bioavailability of 13–23%	Pharmacokinetics Characterization
NHP	Functional Profile of Systemic and Intrathecal Cebranopadol in Nonhuman Primates [72]	Adult rhesus monkeys (*n* = 22)	Subcutaneous cebranopadol	Cebranopadol ED50 2.9 μg/kg [95% CI 1.8–4.6] vs. fentanyl 15.8 μg/kg (≈5.5×); MOP antagonist dose ratio ≈ 9 (dominant); NOP dose ratio ≈ 2 (independent contribution); DOP/KOP: no significant shift; no respiratory rate reduction vs. fentanyl (8 ± 2 breaths/min)	Cebranopadol MOP activation is comparable to fentanyl with lack of respiratory depression and itch, although it showed reduced reinforcing effects.
		Adult rhesus monkeys	Intrathecal cebranopadol (0.18 to 1 μg)	Potent, dose-dependent spinal antinociception without itch sensation; contrast with intrathecal morphine, which produces pruritus as a class effect	These data suggest that cebranopadol could serve as a promising spinal analgesic
Phase 1	Clinical Pharmacokinetic Characteristics of Cebranopadol, a Novel First-in-Class Analgesic [73]	Healthy subjects and chronic pain patients pooled across 14 clinical trials (6 Phase 1 + 2 Phase 1 population PK + 6 Phase 2)	Oral immediate-release Cebranopadol	Tmax 4–6 h; terminal t½ 62–96 h; dose-proportional exposure across clinical range; once-daily dosing pharmacokinetically supported. See published erratum: DOI 10.1007/s40262-018-0686-x	-
	Respiratory Effects of the Nociceptin/Orphanin FQ Peptide and Opioid Receptor Agonist, Cebranopadol, in Healthy Human Volunteers [74]	12 healthy male volunteers	600 μg oral cebranopadol as a single dose	EC50 respiratory depression 62 pg/mL; minimum ventilation >0 L/min (4.9 ± 0.7 L/min)—potential ceiling effect; blood–effect-site t½: respiratory depression 1.2 h vs. analgesia 8.1 h; EC25 antinociception 97 pg/mL	This is a major advantage over full μ-opioid receptor agonists that produces apnea at high concentrations
	Assessment of the Abuse Potential of Cebranopadol in Nondependent Recreational Opioid Users [75]	42 healthy men and women, 18 to 55 years of age, with a history of recreational opioid use	Cebranopadol 200, 400, and 800 μg single oral doses	200–400 μg (therapeutic range): no differentiation from placebo on peak drug-liking VAS (primary endpoint); 800 μg (supratherapeutic): responses comparable to hydromorphone 8 mg, smaller than 16 mg; VAS peak delayed vs. hydromorphone (3 h vs. 1.5 h)	-
	Respiratory and Antinociceptive Effects of Nociceptin Receptor-µ-Opioid Receptor Agonist Cebranopadol versus Full Opioid Receptor Agonist Oxycodone: A Comparison in Healthy Volunteers [69]	30 healthy volunteers	Cebranopadol (600 μg, *n* = 20; 800 μg, *n* = 20; or 1000 μg, *n* = 20)	O_2_ desaturations (to ~80%) in 65% of subjects after oxycodone 60 mg vs. 25% after cebranopadol 1000 μg (2.6× difference); 25% less respiratory depression at model-derived equianalgesic doses of oxycodone vs. cebranopadol in PK-PD analysis.	Most recent paper in the table
Phase 2	Cebranopadol, a novel first-in-class analgesic drug candidate: first experience in patients with chronic low back pain in a randomized clinical trial [65]	Adults with moderate-to-severe chronic low back pain	Cebranopadol (200, 400, and 600 μg given once daily in the morning	All three doses: statistically significant, clinically relevant NRS reduction vs. placebo on both primary endpoints; ≥30% and ≥50% responder rates confirmed; sleep and function improved; higher doses → excess titration-phase TEAEs leading to discontinuations	-
	Cebranopadol: A Novel, First-in-Class, Strong Analgesic: Results from a Randomized Phase IIa Clinical Trial in Postoperative Acute Pain [76]	258 patients who underwent a primary bunionectomy	Single oral administration of cebranopadol 200 μg, 400 μg, or 600 μg	400 and 600 μg > placebo from 2 h to ~22 h post-dose; 400/600 μg outperformed morphine CR 60 mg on primary SPI_2–10_; patient satisfaction higher for 400/600 μg vs. morphine CR; 200 μg = placebo	Connects directly to ALLEVIATE-2 in phase III clinical trials
	Cancer-related chronic pain: Investigation of the novel analgesic drug candidate Cebranopadol in a randomized, double-blind, non-inferiority trial [66]	126 patients with moderate-to-severe pain related to cancer	Cebranopadol single dose vs. morphine PR	Noninferiority AND superiority vs. morphine PR on primary endpoint (FAS: Δ −7.48 mg [95% CI −12.05, −2.92]); ≥75% of patients in either arm had clinically relevant pain reduction; noninferiority NOT met on the key secondary (proportion with clinically relevant pain reduction) TEAEs 83.1% (cebranopadol) vs. 82.0%	-
	Cebranopadol Efficacy and Safety in Diabetic Patients Suffering From Chronic Pain Caused by Damage to the Nerves (NCT01939366) [77]	699 patients with moderate-to-severe chronic pain due to diabetic peripheral neuropathy	Once-daily oral cebranopadol 100, 300, or 600 µg; placebo and pregabalin 600 mg comparator	At week 6, the 600 µg dose significantly reduced average pain vs. placebo (treatment difference −1.01; *p* = 0.0153). At 600 µg, non-serious AEs occurred in 83.9% and SAEs in 6.5%, compared with 69.4% and 3.2% with placebo.	Registry results available; no full peer-reviewed primary publication identified. An additional Phase IIa trial evaluated cebranopadol in diabetic peripheral neuropathy [78]
	Safety and Efficacy Study of GRT6005 in Patients With Osteoarthritis (OA) Knee Pain (NCT01709214) [79]	619 patients with moderate-to-severe chronic pain due to knee osteoarthritis	Once-daily oral GRT6005, 200, 300, or 400 µg or 400, 600, or 800 µg placebo and oxycodone CR comparator	No peer-reviewed efficacy results available.	A separate Phase IIa trial also evaluated GRT6005 in patients with knee OA pain [80]
	Cebranopadol, a Novel First-in-Class Analgesic Drug Candidate: First Experience With Cancer-Related Pain for up to 26 Weeks [67]	Safety and tolerability of prolonged treatment with oral cebranopadol for up to 26 weeks in patients suffering from moderate-to-severe cancer-related pain, who had previously just completed a double-blind trial comparing prolonged-release (PR) morphine twice daily with cebranopadol once daily for six weeks	Cebranopadol doses starting from 200 μg daily titrated up to 1000 μg daily	Safe and well tolerated across 26 weeks; median/mean pain maintained in the mild range throughout; morphine PR → cebranopadol switch safe, well tolerated, and effective for analgesia	-
Phase 3	A Study of Cebranopadol for the Treatment of Acute Pain After Abdominoplasty (ALLEVIATE1) (NCT06545097) [81]	Treatment of moderate-to-severe acute pain after full abdominoplasty in adults	Cebranopadol 400 µg p.o. one hour pre-surgery followed by 400 µg p.o. 24 h after initial dose; another arm used cebranopadol 400 µg p.o. one hour pre-surgery followed by 200 µg p.o. 24 h after initial dose	Primary endpoints met for 400 µg/400 µg arm; significant reduction in pain intensity vs. placebo; 400 µg/200 µg arm did not show significant pain improvement compared to placebo. Rescue medication doses significantly fewer than placebo (for 400 µg/400 µg arm LS mean difference 2.2; *p* < 0.001); tolerability described as comparable to placebo. Nausea was reported as the most common side effect of Cebranopadol	Press release only, pending peer-reviewed publication.
	A Study of Cebranopadol for the Treatment of Acute Pain After Bunionectomy (ALLEVIATE2) (NCT06423703) [82]	Postoperative pain following primary unilateral bunionectomy with first metatarsal osteotomy in adults	Cebranopadol 400 µg p.o. one hour post-surgery followed by 400 µg p.o. 24 h after initial dose	Significant reduction in pain intensity vs. placebo (LS mean difference [SE] 56.1 [13.49]; *p* < 0.001); full efficacy, tolerability, and comparative oxycodone safety data pending peer-reviewed publication.	Press release only, pending peer-reviewed publication.

Comprehensive chronological summary of cebranopadol drug development profile across discovery, in vitro binding/functional assays, rodent and non-human primate models, and Phase I–III human clinical trials evaluating analgesic efficacy, respiratory safety, abuse potential, and pharmacokinetic parameters. It should be noted that, for clinical studies with unpublished or preliminary results, definitive conclusions regarding efficacy and safety cannot be drawn until the complete trial results are formally published.

### 3.3. Translational Pharmacology

The development of Cebranopadol highlights both the opportunities and the challenges of translating NOP receptor pharmacology from experimental research into clinical practice. Translational pharmacology seeks to bridge the gap between molecular discoveries and patient care by combining preclinical pharmacology with clinical investigation to enhance therapeutic outcomes [83,84].

Numerous animal models of acute, inflammatory, and neuropathic pain have demonstrated robust analgesic efficacy [36,38], but the translation of NOP-targeted therapies into human pain management has been less consistent. The discrepancies between preclinical and clinical findings are related to differences in receptor distribution, receptor pharmacology, behavioral pain paradigms, species-specific physiology, and drug metabolism. The interactions between the NOP receptor system and other neurotransmitter networks in the human central nervous system continue to be incompletely understood, necessitating more research into this area [85].

## 4. Functional Implications for Pain and Reward Systems

### 4.1. Pain Modulation Mechanisms

#### 4.1.1. Spinal NOP Activation Produces Antinociception

The nociceptin/orphanin FQ (N/OFQ) receptor (NOP) is widely expressed throughout both the central and peripheral nervous systems. Within the spinal cord, NOP receptors are highly concentrated in the dorsal root ganglia (DRG) and the superficial laminae (I–II) of the spinal dorsal horn (SDH), key regions involved in the processing and transmission of nociceptive information [6,86]. This anatomical distribution enables NOP receptors to regulate pain transmission at an early stage of the ascending nociceptive pathway.

Activation of spinal NOP receptors produces robust antinociception, primarily through Gi/o protein-mediated signaling. Upon N/OFQ binding, NOP receptors inhibit adenylyl cyclase activity, suppress both presynaptic and postsynaptic voltage-gated calcium influx, activate G-protein-gated inwardly rectifying potassium (GIRK) channels, and indirectly decrease sensitization of voltage-dependent sodium channels [6,87]. These coordinated actions hyperpolarize spinal neurons, reduce neuronal excitability, and inhibit the presynaptic release of excitatory neurotransmitters, particularly glutamate and substance P, thereby interrupting the transmission of nociceptive signals to supraspinal pain-processing centers [6,23]. Collectively, these mechanisms produce potent spinal antinociception that resembles the analgesic effects of classical opioid receptors while being mediated through the pharmacologically distinct N/OFQ–NOP receptor system [6,88] (Figure 3).

#### 4.1.2. Supraspinal NOP Activity Modulates Affective Pain Components

Pain consists of two interconnected aspects: a sensory-discriminative element that records the strength and position of a stimulus, and an affective–emotional element that determines the unpleasantness of pain. Spinal NOP receptors primarily modulate sensory nociceptive transmission, whereas supraspinal NOP receptors predominantly regulate the emotional aspects of pain, such as stress, anxiety, fear, depression, and emotional distress. Activation of supraspinal NOP receptors primarily influences the overall pain experience by altering pain-related emotional processing, rather than directly suppressing the transmission of ascending nociceptive signals [6,86].

The main underlying mechanism for these effects is the functional opposition of signaling by corticotropin-releasing factor (CRF), a key component of the extrahypothalamic brain stress system [89]. The central nucleus of the amygdala (CeA) and other limbic regions co-express NOP receptors with CRF pathways, especially CRF1 receptors. Activation by N/OFQ reduces neuronal excitability via Gi/o-dependent signaling (Figure 3), thereby cancelling out the effects of CRF on neuronal activation, without directly opposing CRF receptors [6,89]. Animal studies have consistently demonstrated that intra-CeA administration of N/OFQ reduces anxiety-like behavior in both naïve and stress-exposed animals, highlighting the importance of this pathway in regulating stress-related responses [90,91].

The supraspinal NOP signaling system shows context-dependent regulation of the hypothalamic–pituitary–adrenal (HPA) axis, resulting in an apparent functional dual regulatory pattern. Administration of N/OFQ directly to the central nervous system results in the activation of the hypothalamic–pituitary–adrenal axis, leading to elevated levels of circulating adrenocorticotropic hormone (ACTH) and corticosterone. Activation of NOP receptors within extrahypothalamic limbic circuits reduces CRF signaling, leading to anxiolytic and anti-stress effects system [89,92].

NOP receptors regulate monoaminergic neurotransmission, particularly the noradrenergic system, through mechanisms beyond CRF signaling. N/OFQ consistently suppresses norepinephrine release in neocortical slices and synaptosomes, and activates G-protein-gated inwardly rectifying potassium (GIRK) conductance in neurons of the locus coeruleus, the principal source of central noradrenergic projections [31,92]. The reduction in noradrenergic activity further restricts stress-induced neuronal activation and contributes to the anxiolytic effects of the N/OFQ–NOP system.

Activation of the NOP receptor further influences limbic neuronal networks by reducing both excitatory glutamatergic and inhibitory GABAergic transmission and simultaneously causes neurons to become hyperpolarized through increased potassium conductance [31]. Coordinated actions that occur broadly reduce neuronal excitability within stress-responsive brain regions, in particular the amygdala, leading to an attenuation of fear processing, emotional distress, and pain-related negative affect [6]. Activation of supraspinal NOP receptors collectively demonstrates that alleviation of the affective dimension of pain occurs through the coordinated regulation of limbic stress circuits rather than direct inhibition of ascending nociceptive pathways.

#### 4.1.3. Site- and Species-Dependent Effects of NOP Receptor Activation

Experimental studies have reported both antinociceptive and pronociceptive outcomes depending on the route of administration, agonist dose, species, brain region examined, and the underlying pain model [6,86].

Spinal activation of NOP receptors is consistently antinociceptive, in contrast to supraspinal NOP signaling, which demonstrates a pronociceptive effect in rodent models. These divergent effects reflect differences in neuronal circuitry and the balance between stress, emotional, and descending pain-modulatory pathways across brain regions, despite the mechanisms being incompletely understood.

This divergence is largely species-restricted: in non-human primates, NOP receptor agonists produce antinociception at spinal, supraspinal, and systemic sites with no evidence of the rodent supraspinal pronociceptive (hyperalgesic) phase as shown in Table 5.

Since non-human primate receptor distribution and CNS circuitry more closely approximate the human system, this primate pharmacology has been established as the guiding preclinical framework for the development of systemic dual NOP/MOP agonists such as cebranopadol, with limited evidence and data on site activation effect in humans. Further discussion about the limitation is presented in Section 5.1.1 [6,14].

#### 4.1.4. Integrated Network-Level Regulation of Nociceptive Signaling

The analgesic effects of the N/OFQ–NOP receptor system arise from coordinated regulation of nociceptive processing across multiple levels of the neuraxis rather than from isolated actions at individual anatomical sites [7,10].

Spinal NOP receptor activation suppresses the propagation of nociceptive information through ascending spinal pain pathways and primarily modulates the sensory-discriminative component of pain [6,96].

In contrast, supraspinal NOP receptor activation regulates pain processing within higher brain centers by modulating the affective–emotional dimension of pain.

**Figure 5:**
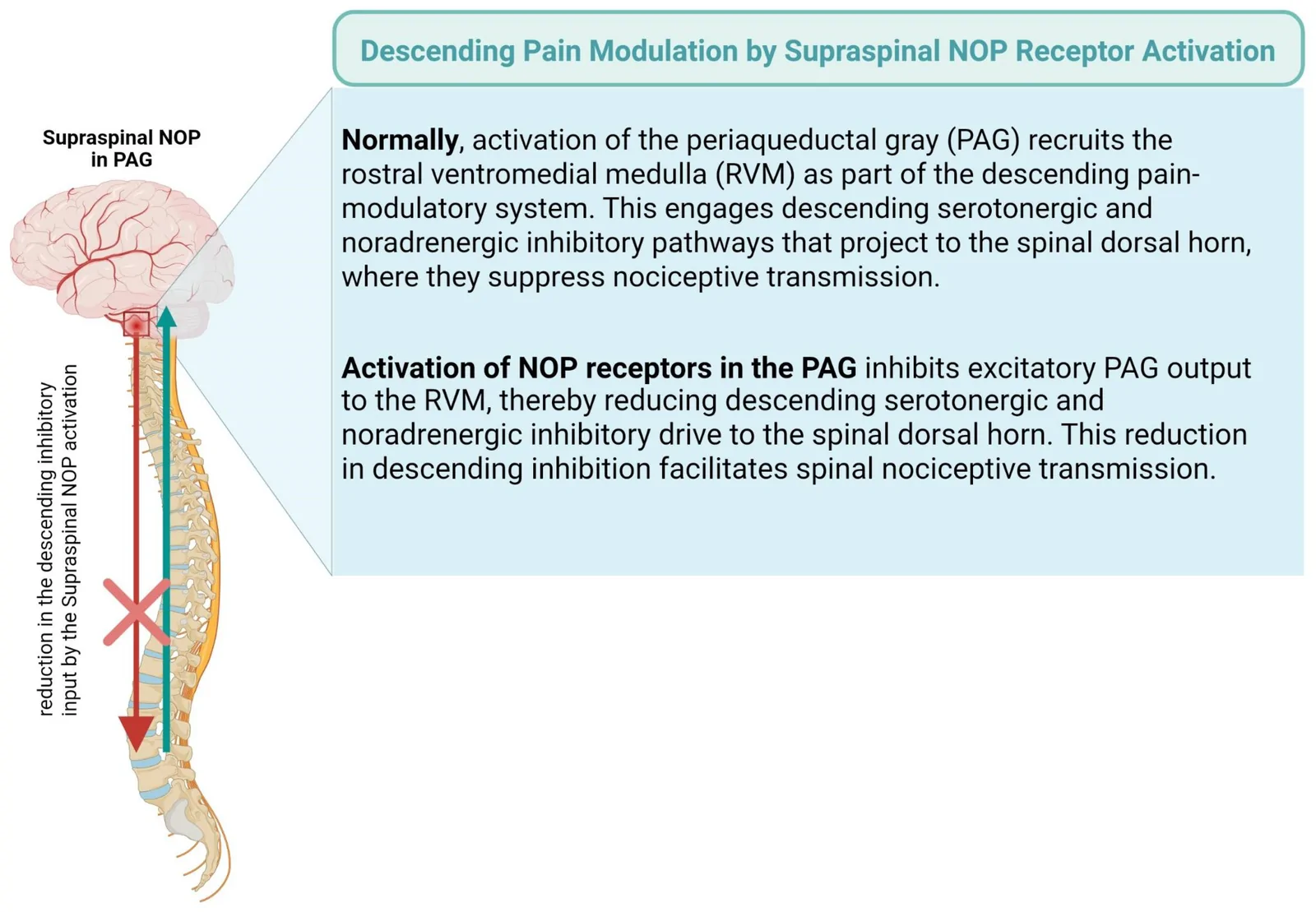
Mechanisms involved in descending pain modulation via supraspinal NOP receptor activation in the PAG. Schematic illustration of supraspinal NOP receptor activation and its role in engaging descending pain modulatory pathways to the spinal cord, resulting in inhibition of transmission. Created in BioRender. Rashed, Z. (2026) https://BioRender.com/muyenq8 (accessed on 30 August 2026).

Experimental studies indicate that NOP receptor activation modulates excitatory projections from the periaqueductal gray (PAG) to the rostral ventromedial medulla (RVM), altering descending serotonergic and noradrenergic inputs to the spinal dorsal horn and ultimately shaping nociceptive transmission [97,98] (Figure 5).

Through this bidirectional interaction between ascending sensory pathways and descending modulatory circuits, the N/OFQ–NOP system integrates sensory, emotional, and motivational aspects of pain into a coordinated analgesic response. This network-level regulation distinguishes NOP receptor signaling from conventional μ-opioid receptor-mediated analgesia by simultaneously providing effective pain relief while suppressing reward-related dopaminergic signaling, thereby offering a mechanistic basis for reduced abuse liability detailed in Section 4.2 [7,10,99].

### 4.2. Reward and Addiction-Related Effects

#### 4.2.1. NOP Signaling Suppresses Mesolimbic Dopamine Activity

The suppression of mesolimbic dopamine activity is mediated through multiple complementary mechanisms. The primary mechanism involves activation of Gi/o-coupled NOP receptors (detailed physiology discussed in Section 2), which suppress dopaminergic neuronal firing, thereby reducing dopamine release from mesolimbic neurons [6,42,86]. Dopamine synthesis is further reduced through inhibition of tyrosine hydroxylase (TH) phosphorylation, resulting in decreased dopamine production [100,101].

Within the ventral tegmental area (VTA), recent circuit-level studies from the Bruchas laboratory demonstrated that Pnoc-expressing neurons located in the paranigral VTA project directly to neighboring dopaminergic neurons. Optogenetic activation of these neurons suppresses reward-seeking behavior by increasing endogenous N/OFQ signaling and reducing dopaminergic neuronal activity [35].

Anatomical evidence further supports local regulation of mesolimbic dopamine by the N/OFQ–NOP system. In situ hybridization studies demonstrated abundant NOP receptor mRNA expression in tyrosine hydroxylase-positive (TH+) dopaminergic neurons, whereas N/OFQ is predominantly synthesized within neighboring GABAergic neurons [102,103]. These observations suggest that endogenous N/OFQ released from local inhibitory neurons acts on adjacent dopaminergic neurons to suppress dopamine neurotransmission, consistent with early pharmacological studies demonstrating reduced extracellular dopamine following NOP receptor activation [87,104].

Within the nucleus accumbens (NAc), NOP receptor activation suppresses dopamine synthesis by inhibiting TH phosphorylation through a cAMP/protein kinase A (PKA)-dependent mechanism. In addition, N/OFQ attenuates dopamine D1 receptor (D1R)-mediated intracellular signaling, including cAMP accumulation, NMDA receptor phosphorylation, and cAMP response element-binding protein (CREB) activation, thereby preferentially suppressing D1 receptor-expressing medium spiny neurons involved in reward learning and reinforcement [100,101,105].

Collectively, these findings demonstrate that NOP receptor activation suppresses mesolimbic dopamine signaling through coordinated regulation at multiple levels of the reward circuitry. By simultaneously inhibiting dopaminergic neuronal firing in the VTA, reducing dopamine synthesis, and attenuating postsynaptic D1 receptor signaling in the NAc, the N/OFQ–NOP system functions as an endogenous negative regulator of reward processing. This integrated mechanism provides a biological basis for the reduced abuse liability of NOP receptor agonists and supports their development as opioid-sparing analgesics [39,42,86].

#### 4.2.2. Potential Reduction in Opioid Reinforcement and Misuse Liability

Drug reinforcement refers to the neurobiological processes that increase the likelihood of repeated drug-taking behavior through activation of the mesolimbic dopamine system. Because NOP receptor activation suppresses dopamine transmission throughout this circuit, it has emerged as an attractive strategy for reducing opioid reward while preserving analgesia [42,86].

Experimental studies demonstrated that intracerebroventricular administration of N/OFQ significantly reduced dopamine concentrations within the nucleus accumbens and attenuated morphine-induced conditioned place preference (CPP), indicating diminished opioid-associated reward learning [104].

Evidence from self-administration paradigms further supports this concept. Cebranopadol, a bifunctional agonist at μ-opioid (MOP) and NOP receptors, produces potent analgesia while reducing cocaine self-administration in rodents. Notably, these anti-addictive effects were abolished following simultaneous blockade of both MOP and NOP receptors, suggesting that coordinated activation of both receptor systems is required for its therapeutic efficacy [6,86].

Genetic studies provide additional confirmation of the physiological role of endogenous NOP signaling. Mice lacking NOP receptors exhibit enhanced sensitivity to opioid reward and increased behavioral responses to drugs of abuse, indicating that the endogenous N/OFQ system normally acts as a negative regulator of reinforcement [86,100].

Although clinical evidence remains limited, available human studies indicate that mixed NOP/MOP agonists produce effective analgesia with substantially lower subjective drug liking, euphoria, and abuse potential than conventional opioid agonists, supporting continued clinical development of NOP-targeted therapeutics [39].

#### 4.2.3. Complex Interaction with MOP-Mediated Reward Pathways

Although NOP receptors belong to the opioid receptor family and share considerable structural homology with μ-opioid peptide (MOP) receptors, the two receptor systems exert fundamentally different effects on mesolimbic dopamine transmission [101].

MOP receptor activation primarily occurs on GABAergic interneurons within the ventral tegmental area, where inhibition of GABA release disinhibits dopaminergic neurons, thereby increasing dopamine release within the nucleus accumbens and reinforcing reward-associated behaviors [35,102,103,104].

In contrast, NOP receptor activation directly suppresses dopaminergic neuronal excitability through the Gi/o-mediated mechanisms detailed in Section 2 and Section 4.2.1. Consequently, NOP receptor activation functionally opposes MOP-mediated reward without abolishing analgesic efficacy [100,101,105].

The two receptor systems also differ in intracellular signaling bias. MOP agonists efficiently recruit β-arrestin-2 and activate downstream MAPK signaling pathways, processes implicated in opioid tolerance, respiratory depression, constipation, and physical dependence [7,87,105]. Conversely, NOP receptor agonists preferentially activate canonical Gi/o signaling while exhibiting reduced β-arrestin recruitment, a pharmacological profile believed to contribute to improved tolerability and reduced adverse effects [42,105].

Another distinctive pharmacological feature of the NOP receptor is its insensitivity to naloxone and other classical opioid antagonists. Despite structural similarity to MOP, DOP, and KOP receptors, naloxone exhibits minimal affinity for NOP receptors even at micromolar concentrations, highlighting the pharmacological uniqueness of the N/OFQ–NOP receptor system [6,39].

### 4.3. Therapeutic Positioning

Instead of assuming greater analgesic potency, the potential clinical value of dual NOP/MOP agonists should be evaluated primarily in terms of the balance between analgesic efficacy and opioid-related adverse effects. Available evidence suggests that the therapeutic effects of NOP-targeted ligands depend on their pharmacological profile and that the clinical usefulness of selective NOP agonism remains incompletely established. Consequently, the rationale for dual NOP/MOP agonists has been to retain substantial MOP-mediated analgesia while potentially reducing some adverse effects associated with conventional MOP agonism [38,106].

Clinical studies of cebranopadol have demonstrated analgesic efficacy across several pain populations, particularly in Phase II trials [65,66]. In patients with chronic low back pain, cebranopadol produced clinically relevant reductions in pain compared with placebo, while a postoperative pain trial demonstrated analgesic efficacy at selected doses and included morphine as an active comparator [65,76]. In cancer-related pain, cebranopadol was shown to be noninferior to prolonged-release morphine and was superior on the prespecified primary endpoint of rescue-medication use, although the trial had substantially lower enrollment than originally planned [66]. These findings demonstrate clinically relevant analgesic activity but do not by themselves establish consistent superiority over conventional opioids across pain conditions. Therefore, an important aspect of its potential therapeutic differentiation is whether clinically meaningful reductions in respiratory depression, drug liking, misuse liability, or other opioid-related adverse effects can be achieved at effective analgesic doses [74,75]. Human studies provide encouraging evidence in this regard; however, cebranopadol can still produce opioid-like respiratory depression, and its safety profile requires evaluation at clinically relevant doses and in broader patient populations [74].

Accordingly, cebranopadol should not currently be considered an established first-line analgesic or a universal substitute for conventional opioids. A potential future role could be as an alternative or opioid-sparing option in selected patients who require effective analgesia but for whom opioid-related adverse effects are an important concern. Possible applications may include selected acute postoperative or chronic pain populations in which respiratory risk, concerns regarding misuse, and the need to reduce supplemental opioid exposure are clinically relevant considerations. However, such positioning would require adequately powered direct comparative trials against standard opioids and non-opioid multimodal analgesic regimens, incorporating clinically relevant outcomes such as analgesic efficacy, respiratory safety, sedation, misuse liability, functional recovery, and long-term tolerability [38,39,106].

#### 4.3.1. Candidate for Opioid-Sparing Analgesic Strategies

Randomized clinical trials indicate that dual NMR agonists are effective for chronic pain management. In a double-blind study of patients with moderate-to-severe cancer-related chronic pain, cebranopadol significantly reduced the need for supplemental opioid medication (Δ −7.48 mg; 95% CI, −12.05 to −2.92) compared to prolonged-release morphine. At least 75% of patients in both groups had significant pain relief, and Cebranopadol was found to be safe and well-tolerated. These results point to a possible opioid-sparing role for cebranopadol in cancer-related pain; however, larger, sufficiently powered comparative studies are needed to confirm the clinical significance of the decrease in supplemental opioid use and its implications for long-term comparative safety [66].

#### 4.3.2. Opportunity for Phenotype-Guided Pain Management

Robust preclinical evidence demonstrates that dual NMR agonists are effective across various pain types. In acute pain, they provide strong, dose-dependent analgesia, as shown in the warm-water tail-withdrawal test in non-human primates. In inflammatory pain models, they reduce pain sensitivity and allodynia, as evidenced by decreased responses in the capsaicin-induced model. Their efficacy is particularly notable in neuropathic pain, where they are approximately 10 times more potent than in standard pain models and outperform conventional MOP agonists.

In addition to analgesia, dual NMR agonists positively influence behaviors associated with opioid use. Preclinical studies show they have lower reinforcement potential than MOP agonists, as indicated by reduced heroin and fentanyl seeking, weaker oxycodone reinforcement, and diminished morphine reward. They also decreased cocaine-seeking behavior in rodent models. Evidence further suggests that NOP receptor activation can mitigate morphine-like dependence, as naloxone reduced dependence-related effects [106].

Another area of interest is use of the N/OFQ system in perioperative care. These agents may be used as adjuncts or alternatives to conventional opioids for managing acute surgical pain, providing effective analgesia with fewer opioid-related side effects. Future research should focus on perioperative and critical-care applications, but there is currently not enough data to support their use for sedation or analgesia in patients on mechanical ventilation. Preservation of spontaneous breathing and maintained responsiveness may support safer patient management, improved neurological monitoring, and faster recovery after sedation [85].

## 5. Challenges and Limitations

Considerable progress has been made in understanding the biology and therapeutic potential of the nociceptin/orphanin FQ (N/OFQ)–NOP receptor system, yet several challenges persist in its successful clinical application. NOP receptor signaling shows a notable dependence on context, with its pharmacological responses differing based on the receptor’s location, the properties of the ligand, the species involved, and the state of the disease. The translation of promising preclinical findings into effective clinical therapies has been hindered by limitations in biomarker development, pharmacokinetic optimization, and long-term safety evaluation. Realizing the full therapeutic potential of NOP receptor-targeted drugs will be essential for addressing these challenges [6,38,86,107].

### 5.1. Mechanistic Complexity

#### 5.1.1. Region-Dependent, Bidirectional Receptor Effects and Their Translational Relevance

**Figure 6:**
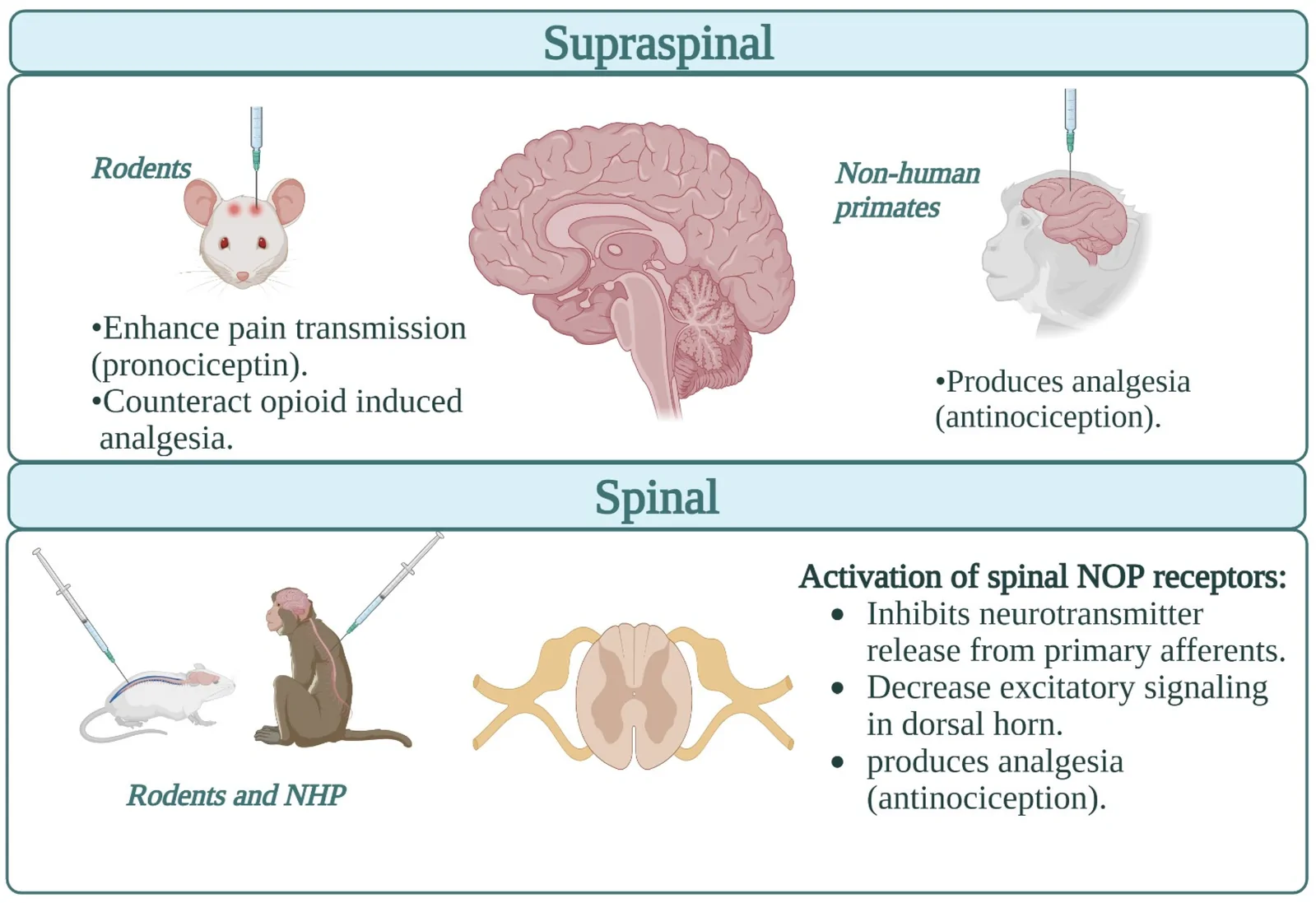
Region-dependent and bidirectional receptor effects. Representation of the bidirectional and distinct effects of NOP receptor activation in the modulation of nociceptive signaling. Created in BioRender. Mohamed, B. (2026) https://BioRender.com/lgc7ysa (accessed on 30 August 2026).

The NOP receptor system is distinguished by its region-specific regulation of pain signaling. Activation of spinal NOP receptors consistently suppresses nociceptive transmission by inhibiting neurotransmitter release from primary afferent neurons and decreasing excitatory signaling within the dorsal horn, leading to substantial antinociceptive effects (Figure 6). In contrast, activating NOP receptors above the spine, especially within the periaqueductal gray, may counteract μ-opioid receptor-mediated pain relief and enhance pain transmission in rodents. Interestingly, this pain-enhancing effect is not consistently observed in non-human primates, where activating NOP receptors systemically or above the spine generally produces pain relief (Figure 6). These findings indicate that the therapeutic outcome of NOP receptor modulation depends on receptor localization, species, and pathological condition, complicating the translation of preclinical findings into clinical practice [6,38,86,96,107].

Findings from non-human primates provide an important translational bridge, but they do not establish that the same receptor interactions will occur in humans or eliminate the uncertainty observed in rodents. In particular, it remains unclear whether the balance between the two receptor systems varies according to dose, receptor occupancy, brain region, pain phenotype, and ligand intrinsic efficacy, or whether NOP activation consistently counteracts MOP-mediated adverse effects in humans [38,96]. Experimental studies of cebranopadol in non-human primates indicate that both NOP and MOP receptors contribute to its antinociceptive effects; in these experiments, selective MOP receptor blockade produced a greater rightward shift in the analgesic dose–response relationship than NOP receptor blockade [72]. These findings suggest that the MOP component may remain an important determinant of analgesic efficacy, whereas NOP activation may modify the overall pharmacological profile of the drug [72].

Thus, although the available evidence supports continued investigation of dual NOP/MOP agonists, it does not justify assuming that NOP activation will automatically counteract all supraspinal or respiratory effects associated with MOP agonism. The translational relevance of the NOP–MOP interaction therefore requires further investigation. Future studies should establish human receptor occupancy, target engagement, dose-exposure relationships, and the respective contributions of NOP and MOP receptors to analgesia and adverse effects. Comparative studies against conventional opioids using clinically relevant endpoints will be particularly important for determining whether the proposed safety advantages are reproducible in patients [72,96,106].

#### 5.1.2. Unclear Contribution of Signaling Bias to Clinical Outcomes

The therapeutic implications of biased signaling at the NOP receptor are still not fully understood. The preference of many NOP agonists for activating G-protein-dependent pathways with relatively weak β-arrestin recruitment has not been clearly linked to analgesic efficacy, receptor desensitization, and adverse-effect profiles. Advances in structural biology and structure-guided ligand design are expected to facilitate the development of ligands with defined signaling bias; however, further experimental and clinical studies are required to determine whether biased agonism translates into meaningful therapeutic advantages in humans [6,107,108].

#### 5.1.3. Difficulty in Predicting System-Level Responses

NOP receptors are widely distributed throughout central and peripheral neural circuits that are involved in pain perception, reward, stress, emotion, cognition, and autonomic regulation. Receptor modulation often leads to interconnected physiological responses rather than isolated pharmacological effects. The crosstalk between NOP receptors and opioid, dopaminergic, glutamatergic, GABAergic, and corticotropin-releasing factor signaling pathways adds to biological complexity and contributes to variability in treatment outcomes. A thorough grasp of interactions at the network level, combined with trusted indicators of target engagement, is therefore necessary to enhance the prediction of therapeutic outcomes and enable personalized NOP receptor-based therapies [6,38,107,109].

### 5.2. Clinical Translation Barriers

#### 5.2.1. Inconsistent Efficacy Across Pain Indications

The clinical efficacy of selective NOP receptor agonists has been inconsistent across different pain conditions, despite their robust analgesic effects in numerous experimental models. The therapeutic response varies depending on the type of pain, the method of administration, the distribution of receptors, the species, and the disease being treated (Table 6). NOP receptor agonists typically show greater effectiveness in long-term inflammatory and neuropathic pain than in sudden nociceptive pain, implying that changes in receptor expression due to disease affect treatment outcomes. These inconsistencies highlight the limitations of current animal models, thereby underscoring the need for more predictive translational models that better replicate human pain mechanisms [86,107,110].

#### 5.2.2. Limited Biomarker Tools for NOP Engagement

The lack of validated biomarkers continues to be a significant barrier to the clinical advancement of NOP receptor-targeted treatments. Preclinical studies have shown biological responses to NOP receptor modulation, as evidenced by several experimental biomarkers, such as alterations in circulating immune cell activation, plasma prolactin concentrations, pharmaco-electroencephalographic responses, and positron emission tomography imaging of receptor occupancy. Currently, none of these methods have been sufficiently validated to reliably quantify receptor engagement or predict therapeutic response in clinical settings. Developing sensitive pharmacodynamic biomarkers that can track target engagement across species is a crucial goal for future translational research [59,110].

#### 5.2.3. Pharmacokinetic and Selectivity Constraints in Drug Design

The development of clinically effective NOP receptor ligands is complicated by challenges in pharmacokinetics and medicinal chemistry. The endogenous peptide N/OFQ has poor metabolic stability, undergoes rapid enzymatic degradation, has limited oral bioavailability, and has inadequate blood–brain barrier penetration, making the development of synthetic ligands with improved drug-like properties necessary. Optimizing non-peptide agonists and antagonists is challenging because several factors, including receptor affinity, selectivity, metabolic stability, CNS penetration, and oral bioavailability, must be balanced simultaneously. Additional complexity arises from dual NMR receptor ligands, whose mixed pharmacology complicates both mechanistic interpretation and clinical optimization [6,63,128].

### 5.3. Safety and Development Concerns

#### 5.3.1. Dose-Limiting Adverse Effects in Some Compounds

**Figure 7:**
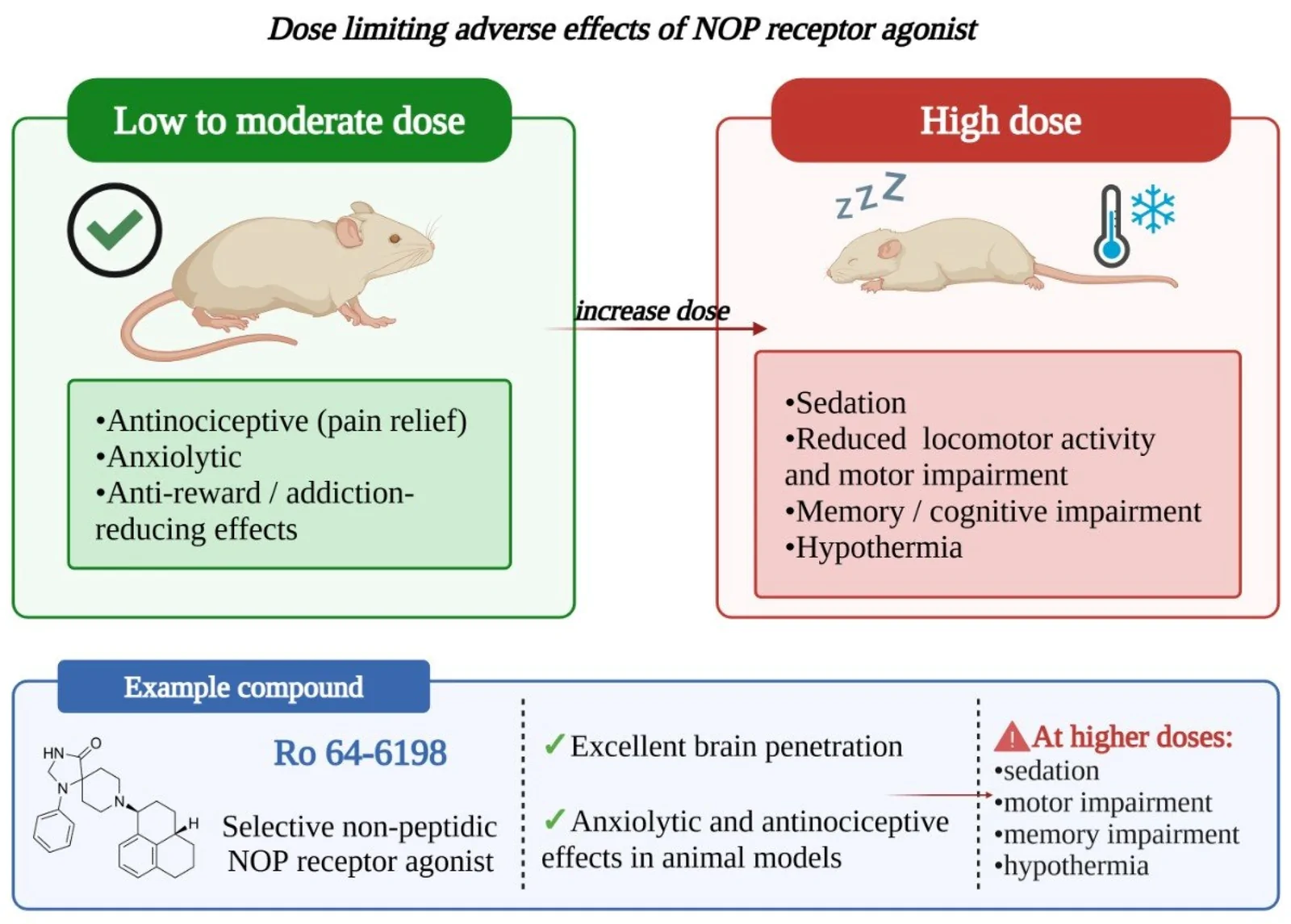
Dose-limiting adverse effects in some compounds. Illustration of dose-dependent effects, showing therapeutic responses at low doses and the emergence of adverse effects at high doses. Created in BioRender. ElSayed, M. (2026) https://BioRender.com/ti5qjeo (accessed on 30 August 2026).

NOP receptor agonists typically cause fewer classical opioid-related side effects, but several compounds exhibit dose-dependent central nervous system toxicity that may restrict their clinical usefulness. Preclinical studies have linked higher doses to sedation, impaired locomotor activity, cognitive dysfunction, and hypothermia (Figure 7). The selective agonist Ro 64-6198 showed promising analgesic and anxiolytic effects, but higher doses led to notable neurological side effects, underscoring the need to optimize the therapeutic window during drug development. Future ligand design should therefore prioritize both efficacy and tolerability through improved receptor selectivity and pharmacodynamic optimization [47,48].

#### 5.3.2. Unresolved Long-Term Tolerance and Adaptation Profiles

The long-term effects of prolonged NOP receptors activation are not yet fully understood. Prolonged receptor stimulation in experimental studies may lead to receptor desensitization and internalization, which could decrease analgesic efficacy over time. Current evidence suggests that NOP receptor agonists produce significantly less physical dependence and withdrawal symptoms than conventional μ-opioid receptor agonists. The exact role of NOP signaling in opioid tolerance remains disputed, as both agonists and antagonists have been found to affect morphine tolerance under various experimental conditions. Subsequently, long-term clinical studies are necessary to clarify receptor adaptation, assess the long-lasting potency of analgesic efficacy, and confirm the safety of sustained NOP receptor modulation [6,47,128].

#### 5.3.3. Regulatory Uncertainty for Mixed-Mechanism Agents

The development of therapeutics targeting the NOP receptor is complicated, especially for bifunctional ligands that activate both NOP and classical opioid receptors at the same time. These compounds must demonstrate not only analgesic efficacy but also a clearly defined contribution of each pharmacological mechanism to both therapeutic and adverse effects, unlike highly selective receptor agonists. Establishing a suitable risk–benefit profile for these medications is considerably more intricate than for conventional analgesics, necessitating a detailed assessment of dual NMR (NOP/MOP) agonism including risk of abuse, respiratory safety, cognitive effects, and long-term tolerance [63,70,128]. Cebranopadol stands out as the most advanced clinical candidate among these agents, exemplifying the opportunities and challenges presented by dual NMR agonism. Studies have shown that cebranopadol offers strong pain relief with reduced respiratory depression and lower risk of abuse compared to conventional μ-opioid receptor agonists [38,48,63,128].

Regulatory agencies are expected to require robust evidence of sustained efficacy, acceptable long-term safety, and reduced misuse liability before approving novel NOP-targeted analgesics. Establishing standardized evaluation frameworks will require close collaboration between academic investigators, pharmaceutical developers, and regulatory authorities to facilitate the translation of innovative NOP receptor-based therapies into clinical practice [38,70,86,128].

It is crucial to remember that safer analgesics are not always more effective. Therefore, the main therapeutic theory behind dual NOP/MOP agonists is that a wider therapeutic window may result in clinically meaningful analgesia rather than necessarily producing more analgesia than traditional opioids. This distinction is crucial because there is currently far more evidence supporting analgesic efficacy and possible safety benefits than superiority in patient-centered clinical outcomes. Future randomized head-to-head trials should ascertain whether the use of dual NOP/MOP agonists over conventional opioid and multimodal analgesic strategies is justified by sufficiently large reductions in respiratory depression, drug liking, or supplemental opioid requirements.

## 6. Conclusions

The N/OFQ–NOP receptor system is a potentially useful, albeit unproven, method for creating new analgesics. Preclinical research, especially in non-human primates, suggests that NOP receptor activation may alter a number of negative effects linked to MOP receptor activation as well as produce antinociceptive and antihypersensitive effects. As a result, dual NOP/MOP agonists like cebranopadol have become significant candidates for translational development.

Nevertheless, there is currently insufficient data to conclude that dual NOP/MOP agonists are more effective analgesics than traditional opioids. Instead, whether clinically significant analgesia can be attained with a more favorable balance between efficacy and opioid-related side effects may determine their potential clinical value. There are still significant uncertainties about dose-dependent adverse effects, long-term safety, comparative effectiveness, and the generalizability of preclinical findings to humans, but available human studies offer encouraging evidence regarding respiratory effects, drug liking, and opioid-sparing potential.

One particularly significant translational challenge is the effects of NOP activation that vary by species and region. NOP agonists typically cause antinociceptive effects in non-human primates, despite the fact that pronociceptive supraspinal effects have been reported in rodents. This distinction encourages more research, but it does not remove any doubt about how the human NOP-MOP system behaves. Furthermore, conclusions about the clinical efficacy of cebranopadol are currently limited due to the lack of complete peer-reviewed primary publications for some recent Phase III findings.

Therefore, long-term safety and misuse surveillance, clinically validated biomarkers of target engagement, adequately powered head-to-head comparisons with conventional opioids and multimodal analgesic strategies, and mechanistic studies that can differentiate the contributions of NOP and MOP activation should be given top priority in future research. Dual NOP/MOP agonists should not be seen as proven substitutes for traditional opioid therapy, but rather as promising investigational analgesics with a potentially distinct safety profile until such evidence becomes available.

## Figures and Tables

**Table 1:** Structural, molecular, and pharmacological comparison between NOP and classical opioid receptors.

Structural Aspect	Key Findings in NOP-Receptor	Classical Opioid Receptors (μ, δ, κ)	Functional Significance
Overall receptor architecture [16,17]	Class A GPCR with seven transmembrane helices (TM1–TM7)	Class A GPCRs with the same seven-transmembrane architecture, extracellular N-terminus and intracellular C-terminus	Confirms membership within the opioid receptor family
Sequence homology [18]	TM2, TM3 and TM7 exhibit >70% conservation; TM1, TM5 and TM6 show ~50% conservation; TM4 is the least conserved domain (24%)	Share high sequence conservation with NOP in TM2, TM3 and TM7; greater similarity among μ, δ and κ receptors than with NOP	Explains structural similarity while preserving receptor-specific properties
Intracellular domains [16]	ICLs are highly conserved, particularly ICL3 (>80%), which links TM5–TM6 and participates in receptor activation and G-protein coupling	ICLs are also highly conserved, especially ICL3, mediating G-protein signaling and receptor regulation	Supports conserved intracellular signaling mechanisms
Activation motifs [17]	Conserved GPCR microswitches are retained in NOP	Conserved GPCR microswitches are similarly preserved in μ, δ and κ receptors	Indicates activation mechanisms broadly shared with Class A GPCRs
Extracellular loops [19]	ECLs are relatively less conserved; ECL2 is enriched in acidic residues, predominantly glutamate, and resembles κ receptors most closely	ECL2 is less acidic and structurally optimized for endogenous opioid peptide recognition	Contributes to receptor specificity
ECL2 function [19]	ECL2 is directly involved in receptor activation and is indispensable for N/OFQ-mediated signaling; supported by chimera studies	ECL2 contributes mainly to ligand binding and receptor stabilization but is less critical for activation than in NOP	Represents a unique activation mechanism among opioid receptors
Ligand-binding pocket [20]	Located mainly within TM3, TM5, TM6 and TM7	Binding pocket also formed primarily by TM3, TM5, TM6 and TM7	Provides the structural basis for ligand recognition
Ligand recognition [6,12]	N/OFQ binds deeply within the pocket; the FGGF motif is essential for binding; Asp130 (D130^3.32^) acts as an anchoring residue	Classical opioid peptides possess the Tyr-Gly-Gly-Phe motif; Asp^3.32^ interacts with the protonated amine of opioid ligands	Stabilizes receptor–ligand interaction
Molecular determinants of selectivity [21]	Only 4–5 amino acid residues confer high selectivity toward N/OFQ and restrict efficient interaction with classical opioid ligands. Despite its structural homology with classical opioid receptors, NOP displays low affinity for conventional opioid ligands and preferentially recognizes nociceptin/orphanin FQ (N/OFQ).	μ, δ and κ receptors exhibit high affinity for endogenous opioid peptides and classical opioid drugs but show poor affinity for N/OFQ	Explains pharmacological distinctiveness
Pharmacological profile [22]	NOP is selectively activated by N/OFQ and exhibits lower constitutive activity than μ and particularly δ receptors	Classical opioid receptors are activated by endorphins, enkephalins, dynorphins and opioid analgesics, and generally display higher constitutive activity	Distinguishes NOP functionally from classical opioid receptors

Summary of the molecular structure, endogenous ligand specificity, receptor selectivity, G-protein coupling, signaling characteristics, and pharmacological properties of the NOP receptor in comparison with classical opioid receptors. These features provide the structural basis for the development of NOP-targeted analgesics with improved safety profiles.

**Table 2:** Functional consequences of NOP receptor activation across neural circuits and relationship to classical opioid receptor systems (μ/δ/κ).

Neural Circuit		NOP Receptor-Mediated Mechanism	Functional Output	Relationship to μ/δ/κ Opioid Systems
**Peripheral**: Dorsal root ganglia (DRG) [28]		Inhibition of N-type Ca^2+^ channels	Reduced sensory neuron excitability and nociceptive transmission	Similar presynaptic inhibitory mechanism through Gi/o-mediated suppression of Ca^2+^ influx
**Spinal** dorsal horn [29]		Reduced C-fiber wind-up, glutamate release, and excitatory postsynaptic potentials (EPSPs)	Attenuation of spinal nociceptive processing	Shared inhibitory signaling mechanisms; however, NOP effects are dependent on pain state
**Spinal** neurons [29]		Inhibition of AMPA- and NMDA-mediated excitatory responses	Reduced excitatory neurotransmission	Similar modulation of excitatory signaling observed with classical opioid receptors
**Supraspinal**	Periaqueductal gray (PAG) [30]	Reduced synaptic transmission (EPSCs and IPSCs)	Modulation of descending pain pathways	Unlike μ-opioid receptors, NOP activation may oppose opioid analgesia in opioid-naïve states
	Dorsal raphe nucleus (DRN) [31]	Activation of GIRK potassium channels	Reduced neuronal activity	Similar inhibitory regulation of neuronal excitability by classical opioid receptors
	Rostral ventromedial medulla (RVM) state-dependent regulation of analgesia [32]	Modulation of pain-related neuronal activity	State-dependent regulation of analgesia	Differs from μ-opioid receptors, which produce more consistent antinociceptive effects
	Hippocampal CA1 region [33]	Inhibition of long-term potentiation (LTP)	Modulation of synaptic plasticity and learning-related processes	NOP receptors exert distinct effects on hippocampal plasticity compared with classical opioid receptors
	Central amygdala [34]	Suppression of glutamatergic transmission and modulation of GABAergic signaling	Regulation of anxiety, stress responses, and emotional behaviors	Both systems influence affective circuits, but NOP has a prominent role in stress-related modulation
	Cholinergic pathways (basal forebrain and brainstem) [33]	Inhibition of acetylcholine release	Modulation of cholinergic neurotransmission, cognitive processing, and autonomic regulation	Similar presynaptic Gi/o-mediated inhibition of neurotransmitter release; cholinergic regulation by NOP receptors is less extensively characterized than that of classical opioid receptors.
	Mesolimbic reward pathway (VTA–nucleus accumbens) [35]	Reduction in dopamine release and reward signaling	Modulation of reward processing and addiction-related behaviors	Opposes μ-opioid receptor effects, as μ activation enhances reward signaling whereas NOP activation generally reduces reward responses
	Nigrostriatal dopaminergic pathway (substantia nigra–striatum) [33]	Reduction in dopamine transmission	Regulation of motor function; relevance to Parkinsonian states	NOP acts as an inhibitory modulator of dopamine pathways, similar to some opioid-mediated dopaminergic regulation

**Table 3:** Pharmacological classification of NOP-active compounds.

NOP Agonist [38]	Mixed NOP/MOP Agonist [38,39]	NOP Antagonist [40]
N/OFQ	Cebranopadol	UFP-101
N/OFQ(1–13)-NH_2_	[Dmt1]N/OFQ(1–13)-NH2	J-113397
[Phe^1^ψ(CH_2_-NH)Gly^2^]N/OFQ(1–13)-NH_2_	PWT2-[Dmt1]N/OFQ(1–13)-NH2	
UFP-112	BU08028	
UFP-113	BU10038	
Ro 64-6198	AT-121	
SCH 221510	SR14150	
MCOPPB	SR16435	
AT-090		
Sunobinop [41]		

**Table 5:** Summary of preclinical NOP receptor modulation across different species and sites.

Site of Activation	Effect in Rodents	Effect in Non-Human Primates
Spinal (intrathecal)	Antinociceptive; enhanced attenuation of hyperalgesia in inflammatory (CFA/carrageenan) models [93]	Antinociceptive—consistent with rodent data [94]
Supraspinal	Pronociceptive (hyperalgesic); low doses can paradoxically worsen inflammatory pain [9,10]	Antinociceptive—no evidence of the rodent hyperalgesic phase [94]
Systemic	Minimal or inconsistent analgesic efficacy [95].	Antinociceptive, without motor or sedative side effects—basis for translational confidence in dual NOP/MOP agonists [95].

**Table 6:** Evidence for the efficacy of different NOP receptor ligands across pain indications and species.

Ligand	Receptor Profile	Acute Pain	Inflammatory Pain	Neuropathic Pain
N/OFQ	Endogenous peptide NOP agonist	**Rodents:** Spinal analgesia; supraspinal pronociception [39,111] **Primates:** Spinal analgesia [112,113]	**Rodents:** Spinal analgesia; supraspinal pronociception [10]	**Rodents:** Spinal analgesia [30]
[Phe^1^ψ(CH_2_–NH)Gly^2^]N/OFQ-(1–13)-NH_2_	Peptide NOP agonist analogue	**Rodents:** Supraspinal pronociception [114]	**Rodents:** Supraspinal pronociception [115]	___
UFP-112	Selective peptide NOP agonist	**Rodents & primates:** Spinal analgesia [115,116]	___	___
Ro64-6198	Non-peptide selective NOP agonist	**Rodents:** Systemic analgesia and occasional pronociception [117] **Primates:** Systemic analgesia [39]	**primates:** Systemic analgesia [118]	**Rodents:** Spinal analgesia [119]
SCH221510	Non-peptide selective NOP agonist	**primates:** Systemic analgesia [50]	**Rodents & primates:** Systemic analgesia [120,121]	**primates:** Reduced capsaicin-induced allodynia [121]
UFP-101	Selective NOP receptor antagonist	**Rodents:** Supraspinal analgesia [122]	**Rodents:** Supraspinal analgesia [123]	**Rodents:** Supraspinal analgesia [123]
[Dmt^1^]N/OFQ(1–13)-NH_2_	Mixed NOP/MOP agonist	**Rodents & primates:** Spinal analgesia [117]	___	___
SR16435	Mixed NOP/MOP agonist	**Rodents:** Systemic analgesia [124]	**Rodents:** Spinal analgesia [125]	**Rodents:** Spinal analgesia [124]
SR14150	Mixed NOP/MOP agonist	___	___	**Rodents:** Systemic analgesia [126]
SR16835	Mixed NOP/MOP Agonist	___	___	**Rodents:** Systemic analgesia [126]
BU08028	Mixed NOP/MOP agonist	**Rodents & primates:** Systemic analgesia [126,127]	**Rodents:** Spinal analgesia [125]	**Rodents:** Spinal analgesia [125]
Cebranopadol	Mixed NOP/MOP agonist	**Rodents:** Systemic analgesia [8]	**Rodents:** Systemic analgesia [8]	**Rodents:** Systemic analgesia [8]

## Data Availability

No new data were created or analyzed in this study. Data sharing is not applicable to this article.

## Abbreviations

ACTH	adrenocorticotropic hormone
AE	adverse event
cAMP	cyclic adenosine mono phosphate
CCI	chronic constriction injury
CeA	the central nucleus of amygdala
CRF	corticotropin-releasing factor
CREB	cAMP response element-binding protein
CGRP	calcitonin gene-related peptide
CPP	conditioned place preference
D1R	dopamine D1 receptor
DOP	δ-opioid peptide receptor
DRG	dorsal root ganglia
DRN	dorsal raphe nucleus
ECL	extracellular loop
EC50	half-maximal effective concentration
ED50	median effective dose
ERK	extracellular signal-regulated kinase
FAS	full analysis set
GABA	gamma-aminobutyric acid
GIRK	G-protein-gated inwardly rectifying potassium channels
GPCR	G-protein-coupled receptor
HPA	hypothalamic–pituitary–adrenal axis
ICL	intracellular loop
i.p.	intraperitoneal
i.v.	intravenous
JNK	c-Jun N-terminal kinase
KOP	κ-opioid peptide receptor
LTP	long-term potentiation
MAPK	mitogen-activated protein kinase
MOP	μ-opioid peptide receptor
N/OFQ	nociceptin/orphanin FQ
NAc	the nucleus accumbens
NMR	nociceptin/orphanin FQ and μ-opioid receptor (NOP/MOP)
NMDA	N-methyl-D-aspartate
NOP	nociceptin opioid peptide receptor
NRS	numeric rating scale
OIRD	opioid-induced respiratory depression
ORL1	opioid receptor-like 1
PAG	periaqueductal gray
PK	pharmacokinetic
PK-PD	pharmacokinetic–pharmacodynamic
PKA	protein kinase A
PNOC	prepronociceptin
PR	prolonged release
RVM	rostral ventromedial medulla
SAE	serious adverse event
SDH	spinal dorsal horn
TEAE	treatment-emergent adverse event
TH	tyrosine hydroxylase
TM	transmembrane
VAS	visual analog scale
VTA	ventral tegmental area
